# Animal Models for Functional Gastrointestinal Disorders

**DOI:** 10.3389/fpsyt.2020.509681

**Published:** 2020-11-11

**Authors:** Alison Accarie, Tim Vanuytsel

**Affiliations:** ^1^Department of Chronic Diseases, Metabolism and Ageing (ChroMetA), Translational Research Center for Gastrointestinal Disorders (TARGID), KU Leuven, Leuven, Belgium; ^2^Department of Gastroenterology and Hepatology, University Hospitals Leuven, Leuven, Belgium

**Keywords:** functional gastrointestinal disorders, animal models, stress, irritable bowel syndrome, functional dyspepsia, intestinal permeability, visceral pain, mast cells

## Abstract

Functional gastrointestinal disorders (FGID), such as functional dyspepsia (FD) and irritable bowel syndrome (IBS) are characterized by chronic abdominal symptoms in the absence of an organic, metabolic or systemic cause that readily explains these complaints. Their pathophysiology is still not fully elucidated and animal models have been of great value to improve the understanding of the complex biological mechanisms. Over the last decades, many animal models have been developed to further unravel FGID pathophysiology and test drug efficacy. In the first part of this review, we focus on stress-related models, starting with the different perinatal stress models, including the stress of the dam, followed by a discussion on neonatal stress such as the maternal separation model. We also describe the most commonly used stress models in adult animals which brought valuable insights on the brain-gut axis in stress-related disorders. In the second part, we focus more on models studying peripheral, i.e., gastrointestinal, mechanisms, either induced by an infection or another inflammatory trigger. In this section, we also introduce more recent models developed around food-related metabolic disorders or food hypersensitivity and allergy. Finally, we introduce models mimicking FGID as a secondary effect of medical interventions and spontaneous models sharing characteristics of GI and anxiety-related disorders. The latter are powerful models for brain-gut axis dysfunction and bring new insights about FGID and their comorbidities such as anxiety and depression.

## Introduction

Functional gastrointestinal disorders (FGID), such as functional dyspepsia (FD) and irritable bowel syndrome (IBS) are highly prevalent, occurring in 10–30% of the general population depending on the criteria used, and represent an important part of the workload of gastroenterology and primary care clinical practice. Those syndromes are characterized by chronic abdominal symptoms in the absence of an organic, metabolic or systemic cause that readily explains these complaints. Based on the Rome IV criteria, FD is defined as bothersome postprandial fullness, early satiation, epigastric pain and/or epigastric burning (1) while IBS is characterized by recurrent abdominal pain or discomfort in association with altered defecation patterns (2, 3). The etiology and pathophysiology remain incompletely understood, which is reflected in the paucity and limited effectiveness of the available treatment options. Moreover, in the last years, FGIDs have been conceptualized as disorders of brain-gut interaction, highlighting the bidirectional interplay between central and peripheral mechanisms and opening new possibilities for anxiety and depression animal models to study FGID.

Over the last 20 years, many animal models, which originally focused on one particular pathophysiological factor, have been developed for FGID. These models have contributed a lot to the understanding of the pathophysiological mechanisms behind the symptoms and were also used to test and validate therapeutic targets and potential therapies (4). However, many aspects of functional disorders remain poorly understood and unsolved in these unidimensional models. An animal model is considered suitable for a disorder, when the etiology of the disease is as close as possible to what is known in humans. Several conditions must be fulfilled, among them (1) the construct validity, i.e., the experimental conditions used to produce the animal model should replicate the cause of the disease in human, (2) the face validity, i.e., the symptoms observed in the animal should replicate the clinical features observed in the patients and (3) the predictive validity where the response to drugs in the animal models can predict reliably the potential response in the human counterpart. It is clear that unidimensional models for FGID do not fulfill these criteria. To overcome the weaknesses of the classic models, more recently, several new models have been introduced, either combining several of the putative factors involved in FGID pathophysiology or using new insights such as the impact of nutrition to try and represent the large panel of symptoms and complex interactions between the pathophysiological factors found in FGID (4).

In this review, we will summarize the state of the art concerning the most relevant and most commonly used pre-clinical models that have been developed for FGID and highlight their contribution to the understanding of FGID pathophysiology. Most of the published studies use rodent models, due to the possibility of genetic manipulations and the quick turnover of the models through fast reproduction and a large number of pups in one litter, in comparison to larger animal models such as pig models. Therefore, this review will largely focus on rodent models.

## Pathophysiology of FGID

Functional gastrointestinal disorders have long been regarded as purely psychosomatic conditions. In the last decade, however, evidence for a low-grade mucosal inflammation, dominated by mast cells, eosinophils, and T-lymphocytes (5–8), as well as impaired epithelial barrier function (9) and neuronal hyperexcitability (10) leading to visceral hypersensitivity (11) and dysmotility has accumulated in FGID, challenging the traditional paradigm of a purely functional disorder. Mast cells represent a crucial link in colonic neuro-immune interaction as they communicate with both the intrinsic; i.e., enteric, and extrinsic nervous system in the gut and release mediators such as tryptase and nerve growth factor, which are involved in visceral hypersensitivity and mucosal permeability in FGID patients (12). A commonly cited hypothesis is based on the concept that luminal antigens, originating from food components, microbiota or other noxious substances such as bile and acid can induce a mast cell and eosinophil predominant immune activation through a failing mucosal barrier which has been found in FD and IBS (13, 14). Nevertheless, it is still unclear whether the mucosal barrier function has any causal role in the pathogenesis of immune activation or whether it is a consequence of the inflammatory response or an unimportant epiphenomenon without a role in disease pathogenesis. Another potentially important player, the microbiota, have been studied intensively in the last years (15). While alterations in colonic and fecal microbiota have been described by several groups in IBS (16), disruption in microbial homeostasis in FD is still largely uncharted territory. Only a limited number of studies have reported alterations in gastric (17) and duodenal microbiota composition in FD (15).

As mentioned above, FGIDs are currently understood as disorders of the brain-gut axis, i.e., the neurohumoral communication system between the brain and the gastrointestinal tract, leading to gastrointestinal hypersensitivity and dysmotility (18). However, central alterations have been mostly studied in the context of visceral hypersensitivity, and the anterior cingulate gyrus, the prefrontal cortex, and the insular cortex have been found to be abnormally activated in IBS patients with visceral hypersensitivity (19). Other structures such as the amygdala or hippocampus have an altered functionality in FGID patients (20). Furthermore, these brain areas are also strongly implicated in psychiatric disorders such as depression and anxiety, two co-morbidities highly represented in FGID patients and associated with visceral hypersensitivity (11, 21, 22).

## Stress-Related Models of FGID

Stress is biologically defined as a physiological response to a stimulus that allows organisms to adapt to their environment (23). However, when stress becomes chronic or occurs whilst important development processes are ongoing, the consequences can be harmful and lead to a predisposition for several diseases, including cardiovascular diseases (24), metabolic disorders (25, 26), depression (27), neurodegenerative diseases (28), drug abuse (29), etc. Over the last decade, the incidence of the stress-related disease has increased, especially in societies where socio-economic pressure plays a crucial role in daily life (30). Physical and psychological stress have been documented intensively as decisive factors in the clinical course of several disorders including FGID (31). The hypothalamus-pituitary-adrenal gland (HPA) axis is the endocrine effector of the stress response, with a central role for corticotropin-releasing hormone (CRH), secreted in the hypothalamus, but also locally in the GI tract (32). By binding to its receptors, CRH stimulates the production and the release of glucocorticoids including cortisol in humans and corticosterone in rodents (33), key regulators of the physiological adaptation to stress (34). In normal conditions, the HPA axis is under rigorous regulation, at both the neuronal and hormonal level, since the glucocorticoid and mineralocorticoid receptors are part of a negative feedback loop which protects organisms against the harmful effect of prolonged exposure to stressors. Nonetheless, a combination of repeated environmental stressors may lead to a maladaptive response resulting in altered brain structure and function (35), predisposing to disease. CRH receptors are found to be expressed in both the GI tract and the central nervous system (CNS) suggesting a crucial role for this factor in the stress-induced disruption of gut homeostasis (36) including transit (37–39), visceral sensitivity (40–42), intestinal permeability (36, 43, 44) and gastric inflammation (45).

Psychological stress and anxiety, often reported by FGID patients, influence the onset of symptoms and predict the clinical outcome (46). Recently, data from our group identified a crucial role for CRH and mast cells in this response, translating previous rodent studies to the human situation (47). Intriguingly, also in inflammatory bowel disease (IBD), longitudinal studies of patients in clinical remission have indicated that stress increases the risk of disease relapse, although the underlying mechanism remains elusive (48). It is still unclear whether stress induces inflammatory changes or whether it is a modulator of symptom perception independent of gut inflammation. Recent studies found that stress influenced the composition of the microbiota, associated with mood disorders and alterations in neurotransmitter pathways (49). Several stress-related animal models have been developed to elucidate the role of stress in the observed changes in the altered sensorimotor function of the gastrointestinal tract in patients with FGID.

In this section, we made a distinction between models of stress applied to adult animals and those involving stress around birth (pre- and post-natal models), i.e., early life stress (ELS). The stress models presented in this section are summarized in Table 1. Evidence from literature indicates that a similar stress paradigm has different effects depending on whether it occurs while the brain is still under development or when the neuronal circuits are already fully developed. Indeed, several studies in humans found that stressors in early life are more likely to result in psychiatric and functional disorders, including FGID (50, 51). Post-natal ELS models use this strategy to induce an increased corticosterone concentration in pups during a period in which they are normally only exposed to low corticosterone levels due to the continuous maternal care (52, 53). The two first weeks of life in rodents (from PND2 til 14) correspond to an insensitive period to environmental stimuli for the HPA axis called stress hyporesponsiveness period (SHRP) (54). During this SHRP, the HPA axis is quiescent and circulating corticosterone, ACTH and CRH levels are very low (55). During this period, stimuli that normally induce corticosterone increase in adults, do not affect pups and the reduced adrenal sensitivity observed is illustrated by the fact that pups do not show a significant elevation of corticosterone concentration following injections of high doses of ACTH (56). The HPA axis maturation mechanisms have not been fully uncovered. Part of these involves enhanced negative feedback due to the low expression of transcortin at the pituitary level (57, 58) and also the decreased expression or transport of hypothalamic secretagogues (59) further supported by the fact that the glucocorticoid regulation of hypothalamic CRH gene expression is not mature during the SHRP (60). Moreover, the regulation of the hypothalamic expression of the arginine vasopressin gene - that occurs at very early stages (61)—which is involved in the ACTH stress response in young rats has a key role for the control of ACTH release from the pituitary (62). Furthermore, it is also the most critical period in the development of central structures such as the amygdala (63) and hippocampus (64). In those two structures, neurodevelopment is very active with neurogenesis, cell differentiation, and migration (65). The existence of such a hyporesponsiveness period suggests that high-stress level might be harmful to the normal development of the brain and could affect the maturation of behavior dependent on those brain systems that are normally developing at that time like the emotional learning systems (57).

**Table 1:** Effect of stress on the Gastrointestinal tract and brain in animal models.

**Stress models**	**Stomach**	**Small intestine**	**Colon**	**Central nervous system**
Prenatal stress			Hypersensitivity (66) Dysbiosis (67) Decreased innervation & increase colonic secretory response to adrenaline (68)	Overactivation of the HPA axis in adulthood with female predominance Epigenetic changes BDNF (SC-female predominance) (67)
Maternal separation	Susceptibility to erosion (83) Delayed gastric emptying (84)		Hyperpermeability during separation At weaning, fecal dysbiosis (74) & hyperpermeability (75) Adult hyperpermeability, mostly transcellular pathway associated with abnormal cholinergic regulation (81) Hypersensitivity (76, 78) Immune cells infiltration (MC Eo) (77) Hypersensitivity of neurons from myenteric plexus to IL6 modulation of secretory and motility functions (79) Increased number of enterochromaffin cells & Increased expression SERT (92)	Decreased activity of glucocorticoid negative feedback (85) increased ACTH response to stressors (86) Increased serotonin concentration in frontal cortex Increased expression 5HT1A/1B/2A in parietal cortex & hippocampus Increased activation of 5HT neuron in raphe magnus and spinal cord (60, 89) Increased sympathetic activity and decreased of parasympathetic activity (94) Increased release of glutamate & AMPA/NMDA receptors involved in remodeling of synapses in hippocampus (95, 96)
Limited bedding			At weaning, fecal dysbiosis (female predominance) & hyperpermeability (69, 74) In adulthood hypersensitivity (male predominance) (102–104)	Decreased social/exploratory behavior & impaired learning and memory (102, 103). Decreased dendritic branching hippocampus Altered thalamo-cortico-amygdala pathway Increased connectivity in locus coeruleus (females) (102) Acts on neuronal development of dentate gyrus (105)
Odor shock conditioning			Hyperpermeability and hypersensitivity through estrogen and GC-C/cGMP pathway; female predominance (104)	Increased expression CRF & GR receptors in central nucleus amygdala (109)
Water Avoidance Stress	Impaired gastric accommodation at D2 via peripheral 5HT2B signaling (119) Increased postprandial gastric contractions at D2 (120)	Hyperpermeability at D1 (112, 113) Structural changes mucus layer at D4 (115) Dysbiosis at D8 (117, 118)	Hyperpermeability at D1 (112, 113) Hypersensitivity at D3 (114) Structural changes mucus layer at D4 (115) Dysbiosis at D10 (117, 118)	Increased glial, neuronal activation in hypothalamus and amygdala & synaptogenesis in the hippocampus (121, 123)
Restrain stress			Increased fecal output CRFR1-dependent manner (37)	Neuronal activation over several brain structures and nuclei including the supraoptic nucleus, locus coeruleus, the ventrolateral medulla, the medial division of the central amygdaloid nucleus, nucleus of the solitary tract and even the dorsal nucleus of the vagus nerve, structures involved in food intake and stress response. Neurons also expressing phoexinin and/or nesfatin (127, 128)
Partial restraint stress	Delayed gastric emptying through sympathetic activation (36, 137) Increased active ghrelin concentration (137, 139, 140)		Hypersensitivity (134–136) Hyperpermeability through re-organization of the cytoskeleton (134) Changes in mucosal morphology and decrease of glial cells in the submucosa plexus Increased immune cells infiltration (MC Eo) (135)	Overactivation of the insular cortex related with colonic hypersensitivity (19)
Crowding stress		Hyperpermeability In WKY rat, activated mast cells (146)	Increased mast cells density (145) In WKY rat activated mast cells, MPO level & transient hyperpermeability, alteration of mitochondrial activity (146)	Anxiety-like and depression-like symptoms Early transient changes (Day 3) in the nitrergic expression in the PFC, hippocampus, hypothalamus (147)
Social isolation			Alterations in the IL-18 pathway and MUC2/TFF3 expression (149)	Anxiety-like and depression-like symptoms Reduced BDNF levels & increased reactivity of the HPA axis (148) Changes in the nitrergic expression in the PFC, hippocampus, hypothalamus (147)
Abdominal surgery	Impaired gastric emptying Low ghrelin concentration (249, 251, 252)	Impaired motility through the activation of the inhibitory reflex pathway increased cytokines expression (TNFa, IL1α, IL6, IL1β, CCL2) (241) infiltration resident macrophages (245)	Impaired motility, delayed transit increased cytokines expression in muscular layers (240) (241) Increased permeability non-related to TLR2/4 (244)	Activation of nuclei (supraoptic nucleus, locus coeruleus, paraventricular nucleus of the hypothalamus & rostral raphe pallidus) expressing nucleobindin2/nefastin complex involved in the decrease of food intake and GI transit (253)

*5HT, serotonin; 5HT1A/1B/2A/2B, serotonin receptor type 1A, 1B, 2A, 2B; ACTH, adreno cortico trophic hormone; AMPA, α-amino-3-hydroxy-5-methyl-4-isoxazolepropionic acid; BDNF, brain-derived neurotrophic factor; CCL2, chemokine ligand 2; CRH, corticotropin-releasing hormone; CRHR1 corticotropin-releasing hormone receptor type 1; D1/2/3/4/8/10, day 1/2/3/4/8/10; Eo, eosinophils; GC-C/cGMP, guanylate cyclase C/cyclic guanylin monophosphate; GI, Gastrointestinal; GR, Glucocorticoid receptor; HPA, hypothalamus-pituitary-adrenal; IL, interleukin; MC, mast cell; MPO, myeloperoxidase activity; MUC2, Mucin 2; NMDA, N-methyl-D-aspartate; PFC, prefrontal cortex; SC, spinal cord; SERT, serotonin transporter; TFF3, rail fold factor 3; WKY, wistar kyoto*.

### Prenatal Stress

In the last decade, data coming from clinical psychiatry showed that women's health status before and during pregnancy is a determining factor for the development of psychiatric disorders, including schizophrenia and depression, socio-emotional problems or altered stress response of the children later in life (49). History of a poor socio-economic context, malnutrition, obesity, metabolic disease, depression, and anxiety in the mother, has been linked to the development of FGID in the child (51). Several paradigms have been used in rodent mothers, including repeated daily immobilization, exposure to noise, sleep deprivation or an alternation of unpredictable stressors to mimic the human situation (49). The effect of those maternal stressors on the development of psychiatric disorders in the offspring is well described, and their effect on the GI tract have been studied in a couple of them. Winston et al. demonstrated that unpredictable chronic stress, i.e., a random sequence of twice daily application of either water avoidance stress, cold restraint stress or forced swimming stress, applied from mid-gestation until delivery in pregnant Sprague Dawley rats induced colonic hypersensitivity in both male and female offspring. Besides, when the animals were re-exposed to the same pattern of stress as their dam at 8–12 weeks of age, they displayed an increased response compared to the offspring of non-stressed dams with a more pronounced effect in females (66). The observed effect in females correlated with epigenetic modifications of the brain-derived neurotrophic factor (BDNF) gene in the dorsal horn of the lumbosacral spinal cord. Using the same stress model in mice, Jasnarevic et al. could point out a strong link between the stress-altered maternal microbiota and neurodevelopmental alterations and microbiota composition in the offspring (67). This important finding was further confirmed in another study that demonstrated long-lasting changes in the intestinal microbiota composition, associated with a deficiency in the innervation of the distal colon and an increased colonic secretory response to adrenergic stimulation and an exaggerated response of the HPA axis to stress (68).

### Neonatal Stress

#### Maternal Separation

The most frequently studied and used model of ELS is the maternal separation (MS) model in which pups are separated from the dam and the rest of the nest every day during their first weeks of life. Multiple variations of the MS procedure have been described, with changes in the duration of the separation and the number of days of separation. Variations include MS until weaning whereas other protocols use a 24 h separation between post-natal day 3 (PND3) and PND4 (69). The most common protocol consists of about 12 days of separation, 3 h per day, starting from PND2 till PND14. This daily separation induces anxiety in the mother (70) leading to a discontinuity in maternal care and an abnormal mother-pups relationship (70, 71) and initiates the premature end of the hypo-responsiveness period through a rise of corticosterone in the pups (72). Short separation (< 60 min) is not harmful and separation of 15 min even diminishes anxiety-related behavior and the pup's response to stress later in life (73). Repetitive, short separations are reminiscent of the natural behavior of the mother who needs to gather food. However, longer separation mirrors caregivers neglect and physical and psychological abuse during childhood.

Pups who underwent the maternal separation, display an increased intestinal permeability as well as changes in the microbiota composition at the time of weaning which are associated with an increase in the basal level of corticosterone (74). Changes in the microbiota included a lower diversity of the microbiome with a decrease of the fiber-digesting bacteria, mucus-resident and butyrate-producing bacteria (74). Another study showed that, during the maternal separation (at PND9) in mice, the separated pups had an enhanced permeability with a decreased trans-epithelial electrical resistance and an increased transcellular permeability in the colon while the small intestine was not affected (75). In adulthood, at 2 to 3 months, animals who underwent the maternal separation protocol (12 days of separation 3 h/day) have an increased response to colorectal distension, which was more pronounced in mice than in rats (76). In fact, in rats, the MS protocol alone does not change visceral sensitivity but rather induces a susceptibility to develop visceral hypersensitivity when animals are re-exposed to an acute stressor later on during adulthood. Interestingly, this susceptibility is transmitted across generations through a mast cell-dependent mechanism (77). The latter is an important part of the immune cell infiltration characterized after MS in the intestinal mucosa. When activated, mast cells released mediators such as histamine and other inflammatory factors including IL6 and nerve growth factor (NGF), which are able to sensitize the nerve endings located in the colonic mucosa which express ionic channels including the transient receptor potentiate vanilloid 1 (TRPV1). The modulation of this ion channel has been shown to be an important factor in the maternal separation stress-induced visceral hypersensitivity (78). O'Malley et al. also demonstrated an IL6-dependent hypersensitivity of the neurons from the submucous plexus, which are involved in the secretory and motility function of the colon (79). Local inflammatory mediators such as the myeloperoxidase activity (MPO), IL4, IL1β, or IFNγ are also associated with intestinal barrier dysfunction and to an alteration in morphology of the colon of adult rats (80). The increased permeability described in maternally deprived animals mainly involves the transcellular pathway and seems to involve an abnormal cholinergic regulation of the epithelial permeability (81). As previously mentioned, CRH receptors are expressed along the GI tract and CRH is one of the mediators of the GI effects of maternal separation. However, the two receptors for CRH have a differential effect on the intestinal physiology. Indeed, while maternally separated adult rats treated with CRH Receptor1 (CRHR1) antagonists displayed a decreased inflammation, the group treated with a CRH Receptor2 (CRHR2) antagonist showed an inhibited stem cell activity and injury repair. CRHR1 contributes to intestinal injury and modulation of the microbiota while CRHR2 promotes healing and repair of the intestine (82). Besides the well-documented colonic injury in animals submitted to maternal separation, studies also showed an alteration in gastric function characterized by enhanced susceptibility to gastric erosion (83) and a delayed gastric emptying associated with structural changes in the glial cells (84).

The separated pups develop an increased reactivity of the HPA axis in response to stress during adulthood (71), as shown by a decreased activity of the glucocorticoid negative feedback loop (85) and an increased adreno cortico trophic hormone (ACTH) response to a stressor (86). Several central neurotransmitter pathways are also affected by MS including the serotonergic (87), cholinergic (81) and glutamatergic (88) pathway. The serotoninergic pathway is altered with an increased 5-hydroxytryptamine (5HT, serotonin) concentration in the frontal cortex and increased expression of the 5HT 1A, 1B, and 2A receptors in the parietal cortex and the hippocampus (60, 89). Furthermore, MS rats showed increased activation of serotoninergic neurons in the raphe nucleus and the spinal cord. Rat studies have demonstrated the involvement of the 5-HT1A receptor in the pathophysiology of stress-induced visceral hypersensitivity as a treatment with the mast cell blocker Resveratrol was potentiated by a pre-treatment with a 5-HT1A agonist (90). In the same way, a therapeutic effect of anti-depressants targeting the monoaminergic system has been reported in this model (91). At the enteric nervous system (ENS) level, an increased number of enterochromaffin cells producing 5HT and increased expression of the serotonin transporter (SERT) were observed in MS rats (92). These findings may contribute to the observed altered sensorimotor function in FGID patients with a childhood abuse history, although the role of enterochromaffin cells and SERT has not been studied in particular context. Increased noradrenaline levels, the main neurotransmitter of the sympathetic branch of the autonomic nervous system, in the cingulate cortex was associated with fear and anxiety in the MS model (93). In IBS patients, studies measuring heart rate variability confirmed an increased sympathetic nervous system activity and a decreased parasympathetic nervous system activity (94). Alterations have also been described in the glutamergic pathway, which is involved in emotion and cognitive behavior. Maternal separation induced a release of glutamate in the hippocampus which activated receptors leading to neuronal excitotoxicity (95). In the hippocampus of the MS rat, an increased expression of the α-amino-3-hydroxy-5-methyl-4-isoxazolepropionic acid (AMPA) and the N-methyl-D-aspartate (NMDA) receptors have been found to be associated with a remodeling of the synaptic plasticity (95, 96). Alterations in the hippocampus also concerns neurogenesis which explains the long-term consequences in adult behavior. Maternal separation affects the neurogenes which is very active during the SHRP and leads to impaired coping behavior in adulthood and the learning process (97).

#### Limited Bedding

As mentioned above, the socio-economic status of caregivers affects the onset of FGID in children. To mimic poverty and precarious conditions in humans and also to limit the intervention of an external experimenter which is subject to variability, a model of limited cage bedding, mostly applied between the PND2 and PND9, was developed first in rats and later also in mice (98, 99). Some recent studies have proposed a variation by applying intermittent limited bedding from PND1 to PND7 or limited bedding from PND8 to PND12 (100). In this model, the female does not have access to any form of enrichment to build a nest. This altered environment is stressful for the mother and leads to a fragmentation of maternal care without changing the overall duration. The periods of maternal care are shorter and the behavior is more frequently shifted from one to another e.g., grooming, nursing, going in or out of the nest, self-licking, self-grooming… (101). The mother's stress can be modulated by varying the amount of nesting material introduced in the cage. With this protocol, the stress applied to the pups is chronic, unpredictable and uncontrollable which has good construct validity for stress-related disruption of parental care in a context of economic difficulties (69). The profound chronic stress induced by this protocol leads to a transient increase of the corticosterone concentration and hypertrophy of the adrenal glands, increased intestinal permeability and a fecal dysbiosis in 21-day old pups while these effects had disappeared at 12 months (69, 74). Although the concentration of corticosterone was strongly correlated with the hypertrophy of the adrenal glands, the elevated concentration of corticosterone was associated neither with the observed dysbiosis (lower diversity and increased abundance of genera of Gram positive cocci) nor intestinal permeability, for which a sex difference was observed with the females being more affected than the males (74). However, in adulthood, the rats submitted to this early life stress showed a reduction in social and exploratory behavior, impaired learning and memory processes, a decreased dendritic branching in the hippocampus, an increased response to a stress challenge and visceral hypersensitivity (102, 103). The latter, depending on the method used for the colonic pain assessment, showed no sex difference or a higher colonic sensitivity in males (104). Similar results were observed for anxiety-like behavior. Moreover, differences in the brain connectivity of the thalamo-cortico-amygdala pathway during a painful stimulus have been reported to be altered in rats submitted to this protocol of ELS. The authors pointed out a sex difference with increased brain connectivity in the locus coeruleus/lateral parabrachial nucleus in females only (102). A recent finding showed that limited bedding affects the neuronal development of the dentate gyrus and depletes the stem cell pool in adult animals but does not influence neurogenesis (105).

#### Odor Shock Conditioning

Many mammals including rodents are born blind and deaf, and to stay warm and obtain the food and care needed to survive, they need to learn the odor of their caregiver, e.g., their mother. For this purpose, during the first 9 days of their life, pups display an enhanced capacity for odor preference learning through a stimulated release of norepinephrine, produced in the locus coeruleus, which binds to its receptors in the olfactory bulb (53). This allows the pups to learn the odor of their caregivers without associating it to fear or aversion. Furthermore, during this period, the pups display an inability to initiate a stress response. Once this period ends, the level of secreted norepinephrine decreases, associated with the development of the α2 inhibitory auto-receptors functionality and the downregulation of the α1 excitatory auto-receptors (53). In a second phase, called the conditional sensitive period, from PND10 till PND15, pups start to explore their environment and learn avoidance and fear for aversive stimuli in the absence of the mother. As we described earlier, the maturation of the HPA axis and maturation of fear behavior happens during the time-window in which neuronal circuits are located on the trajectory of the HPA axis maturation. Several studies showed that the rise of corticosterone levels starts during this period and is critical for the engagement of the amygdala and the learning of aversion and fear in response to a stimulus such as the odor of a predator (106). Interestingly, during this period, the presence of the mother can reengage the fear learning process (107).

Developed by and mostly used in the Greenwood-Van Meerveld lab, the model of odor shock-conditioning consists of predictable and unpredictable odor-shock pairings which mimic attachment to an abusive caregiver. This model uses the association between an odor and a modest electrical shock to the tail to reproduce the pup-dam interaction and creates an olfactory attachment to the conditioned odor in response to predictable or paired odor/shock. In practice, pups are exposed from PND8 to PND12 to an odor associated with an electrical shock 2 minutes after the odor exposition while controls are only exposed to the odor (104, 107–109). During adulthood, only female Long-Evans rats displayed an increased colonic permeability and a colonic hypersensitivity which persisted later in life and seemed to be directly linked to estrogen concentrations, as an ovariectomy in females subjected to the odor shock conditioning model, rescued this phenotype (108). Moreover, increased expression of the CRF and the glucocorticoid receptor was found in the central nucleus of the amygdala which was involved in the maintenance of the colonic hypersensitivity (104). The use of linaclotide, a guanylate cyclase C (GC-C) agonist used in clinical practice to treat constipation-predominant IBS, restored both colonic hypersensitivity and permeability, proposing the GC-C/cGMP pathway as an important player in the peripheral regulation of the persistent visceral pain in adults exposed to this form of ELS (109).

### Adult Stress

#### Physical Stress

Water avoidance stress (WAS) is one of the most frequently used models of stress in adult rodents either alone or combined with the maternal separation model. Several studies over the last decade have used it to characterize acute and chronic stress-induced GI symptoms and to study the effect of treatment, including nutritional and probiotic interventions (110, 111). During this protocol, the animal is placed on a platform (usually 10 × 10 for rats and 3 × 3 cm for mice) surrounded by water, either cold or at room temperature, until 1 cm below the platform. The water reservoir should be large enough to avoid the animal to jump out and thus give the animal the impression that no escape is possible. This protocol can serve as either an acute or a chronic stressor and mimics resilience to an uncomfortable situation. A large number of variations on the protocol have been described in the literature and often differ by their duration. WAS induces a robust activation of the HPA axis which transiently alters gut physiology (110). One day of stress induces an increased intestinal permeability (112, 113) while 3 days of stress are needed for colonic hypersensitivity to appear (114). Morphological changes, including the composition and the structure of the mucus layer, were present from the fourth day (115). Interestingly, the follicle-associated epithelium in the ileum seemed to be more affected than the colon (116). A fecal dysbiosis, including an altered composition and function of the microbiota, has been described after 10 days of stress in rats while in mice it already appears after 8 days in the small intestine (117, 118). Gastric contractions after a meal were increased in rats after two sessions of WAS, through the activation of the peripheral CRH1 receptors (119). Impaired gastric accommodation occurred after 2 days of stress and was mediated through the peripheral serotoninergic receptors 5HT2B (120). In mice, after four sessions, alterations in the brain occurred with an increase of the neuronal and glial activation in the hypothalamus, hippocampus, and amygdala. These structures are not only involved in the stress response but also in memory, pain and emotion pathways which are often found to be altered in IBS patients (121–123). A sex-difference has been described in the processing of emotional signals in healthy humans (124), in patients with FGID (125) and stress-related visceral hypersensitivity in rats (126).

As for the maternal separation, several models of physical constraint have been developed over the years, among them three versions of physical constraint: the partial restraint stress, full restraint stress and cold restraint stress. The extent and the duration of the stressor differ amongst the protocols. With a full body restraint stress applied in rats for 30 min, studies have shown neuronal activation over several brain structures and nuclei including the supraoptic nucleus, locus coeruleus, the ventrolateral medulla, the medial division of the central amygdaloid nucleus, nucleus of the solitary tract and even the dorsal nucleus of the vagus nerve, structures involved in food intake and the stress response (127, 128). Interestingly, in those nuclei and structures, the activated neurons also expressed nesfatin-1 and/or phoenixin, two peptides involved in the regulation of food intake and anxiety behavior (129, 130). Nesfatin-1 is mainly expressed in the hypothalamus and brainstem where it colocalized with CRF (131). When administered directly into mice brain, nesfatin-1 led to an increase of plasmatic ACTH and corticosterone levels, as well as an activation of neurons expressing CRH, noradrenalin and serotonin, indicating both a central and a peripheral response to stress. Furthermore, the nesfatin-1 system is activated when rats are submitted to restraint stress for 1 h (132). In mice, a protocol of 60 min of full restraint stress showed a CRF-dependent increase of pellet output which was abolished by central injection of a specific CRFR1 antagonist—while a CRFR2 specific antagonist had no effect—(37) and also by a systemic injection of peptide YY (133). Partial restraint stress is another common form of the model which consists of restriction of the upper body movements. In this model, the shoulders, upper forelimbs, and thoracic trunk of the animal are wrapped in a confining harness of paper tape or cloth to restrict, but not to prevent, body movements (134). This protocol is mostly used as an acute stressor with a 1 to 2 h period of restraint. However, this short exposure already promoted (1) colonic hypersensitivity (134–136), (2) an increased influx of immune cells in the mucosa, mostly consisting of mast cells and eosinophils (135), (3) an intestinal hyperpermeability through the reorganization of the cytoskeleton in epithelial cells (134), (4) a delayed gastric emptying associated with the stress-induced sympathetic activation, increased CRF (36, 137) and associated peptides (138) as well as active ghrelin concentration (137, 139, 140), and (5) changes in colonic morphology and a decrease of enteric glial cells especially in the submucosa plexus (135). By using this model for 14 days, Yi et al. could demonstrate the implication of the insular cortex in stress-induced visceral hypersensitivity, a region found to be abnormally activated in FGID patients (19) and more in general in patients with chronic pain (141).

#### Social Stress

One of the important findings of the last century in the field of psychobiology is the stress-buffering effect of social relationships, with an important role for oxytocin (142). Social buffering conceptualizes the idea that social support can attenuate the stress response and reduce the release of stress hormones (143). As we discussed in the previous section, the more powerful demonstration of this concept is the mother's social buffering of the offspring in which the mother and the pups can influence each other's corticosterone concentration (144).

However, the positive effect of this social buffering depends on the nature of the relationship between individuals as well as on the social organization of the species and/or gender. Many species, including humans and rodents, live for almost their entire life in a group with a strong hierarchy. As a result, any disturbance in this social order or abuse is a potential source of stress. In rodents, stress models used either the isolation of one animal from the rest of the group, i.e., social isolation stress, or, at the other end of the spectrum, an overpopulation within a small area, i.e., crowding stress. Often used as models for anxiety and depression-related disorders alterations of the GI physiology have also been studied in rodents submitted either to the social isolation stress or the crowding stress models. Crowding induces a strong competition for space, food, and water and leads to a strong increase of the corticosterone concentration in the first days associated with early transient alterations in the nitrergic system in the hippocampus, prefrontal cortex and hypothalamus, an increase of iNOS expression in all structures and increased of nNOS in the hippocampus and hypothalamus. These changes are normalized over time through a habituation process but remains higher than in normal housing conditions. This protocol is used for 2 to 9 weeks depending on the strain and type of rodent and is often combined with other types of a stressor to simulate the combination of chronic and (sub)acute stressors that naturally occurs in humans. Work from our group demonstrated that 14 days of crowding stress in Wistar rats induced increased permeability in the jejunum which correlated with plasma corticosterone levels. However, mast cell density was only increased in the colon (145). In Wistar Kyoto rats, a strain sensitive to anxiety, a crowding stress protocol applied for 15 days induced a transient increase of the small intestine and colon permeability associated with a transient rise of MPO activity and altered mitochondrial activity (146) as well as mast cell infiltration (colon) and activation in the GI tract (small intestine and colon) (146).

Conversely, in the social isolation stress (SIS) model, the animal is isolated from the rest of the cage. Often applied just after the weaning, SIS modifies the development of the brain and influences the nitrergic system in several brain areas such as the hippocampus, the frontal cortex by increasing the nNOS expression in the hippocampus and hypothalamus and iNOS in the prefrontal cortex (147). A decrease production of BDNF, and an activation of the HPA axis which produce more corticosterone. Mice exposed to SIS, have an impaired reactivity to stress with an overreaction to another stressor together with increased anxiety- and depression-like symptoms (148). In the GI tract, regional differences have been pointed out between the colon and the rectum concerning MUC2/TFF3 expression and in the IL-18 pathway in mice exposed to 16 days of SIS (149).

#### Combined Stress and Chronic Mild Unpredictable Stress

Although the various animal stress models developed over the last years have provided critical information about the influence of stress on physiology, humans are usually not exposed to only one stressor during their life and a combination of stressors is often present in patients with FGID. Also, physiology can adapt to one homotypic stressor in humans and rodents, leading to habituation and absence of effect after repeated exposure. As a variety of animal models of stress are available, a wide range of stress combinations can be used to better understand the pathophysiological mechanisms behind stress-induced FGID symptoms. Combinations of unpredictable mild stress are also often used with a rotation between different stressors such as light/dark cycle, isolation, crowding, predator odor exposure, shock, cold environment, restraint stress….

An often-used combination in rats is the maternal separation combined with one session of water avoidance stress in adult rats. In contrast to mice, maternal separation is not always sufficient to induce GI symptoms in rats (76) but increases the susceptibility for GI symptoms upon subsequent exposure to stress (150) which is transmitted to the next generation (77). In another type of combination of early life stress, i.e., odor shock conditioning, and water avoidance, a sex-difference was observed with a more pronounced female susceptibility to develop visceral hypersensitivity, which is in line with the female predominance in FGID (151).

With the use of unpredictable stress models, which consist of applying a stressor (SIS, restrain, WAS…) at unpredictable moments of the day for a few days, the involvement of nerve growth factor, endorphin, beta-adrenergic pathway, BDNF and mast cells mediators and the toll-like receptor 4 (TLR4) pathways have been demonstrated in stress-induced visceral hypersensitivity (107). Recent studies suggested a role of an altered microbiota in the anxiety and depression-like behavior in animals exposed to unpredictable mild stress with a strong correlation between the alterations in the microbiota and colonic serotonin concentrations (152, 153).

## Low Grade Inflammatory, Post-Infectious and Post-Inflammatory Models of FGID

Infections and inflammation are among the best-characterized triggers for FGID symptoms. Although the pathophysiological mechanisms are not yet fully understood, a low-grade inflammation is considered as the main explanation for the symptoms in so-called post-infectious (PI) IBS and FD (154). Evidence from IBD patients in remission has also brought some more arguments for this mechanism of persistent low-grade inflammation triggering IBS-like symptoms (155). Psychological factors have been shown to be associated with the prevalence of PI-IBS as well as somatization which, when happening during the infectious period, is positively correlated with the incidence of IBS symptoms (156).

### Low Grade Inflammatory Models

Low dose injections of inflammatory factors like the bacteria-derived lipopolysaccharide (LPS), injected systemically at the dose of 1 mg/kg can also trigger FGID features such as rectal allodynia and colonic hyperpermeability. Visceral allodynia appears 3 h after injection last up to 12 h and is mediated by mast cell degranulation, IL1β and TNFa (157). When Authors performed a subdiaphragmatic vagotomy, they observed an increased allodynia compared to sham animal which suggest that the rectal allodynia seen after LPS injection is controlled by the vagus nerve (158). In another study, both the hyperpermeability and allodynia observed 3 h after injection, were normalized with antagonist of TLR4 and IL1β as well as with a CRFR2 specific agonist and Astressin a non-selective CRFRs antagonist suggesting an important role of the CRF in the effect of LPS injection on visceral sensitivity and permeability (159). Moreover, allodynia and hyperpermeability were abolished with peripheral injection of a selective CRFR2 agonist and with an non-selective inhibitor of CRF receptors (157). Losartan, an angiotensin II blocker, and lovastatin reversed the permeability and allodynia, dependent on the macrophage peroxisome proliferator-activated receptor gamma (PPARγ) and the endogenous opioid, dopaminergic and nitrergic system, potentially opening the door for novel therapeutic strategies of FGID (160, 161). Similarly, the tricyclic antidepressant imipramine reversed LPS-induced allodynia and colonic hyperpermeability (162).

Several models have also been developed using a low concentration of dextran sodium sulfate (DSS) to induce low-grade inflammatory changes (163). This model contrasts with the post-inflammatory model of high-dose DSS followed by a recovery period, discussed in the next paragraph. An overexpression of the T-type calcium channel cav3.2 in this model, which was also observed in colonic biopsies of IBS patients, was associated with colonic hypersensitivity in this model (163, 164).

### Post-inflammatory Models

Rodent models of post-inflammatory GI disorders attempt to simulate a post-inflammatory situation mostly represented by the resolution of an acute infection. Most of the models presented below originally are models for IBD but can be instrumental to study certain aspects of the development of GI symptoms after resolution of the acute inflammation. The development of post-inflammatory GI disorders occurs in 25 to 100% of the treated animals depending on the trigger used. Furthermore, the severity of symptoms and functional alterations developed in the post-inflammatory period—except for the visceral hypersensitivity—seems to be independent of the severity of the initial inflammation (165).

An acute treatment with a high percentage of DSS followed by a DSS-free period (166) creates a remaining low-grade inflammation which is associated with visceral hypersensitivity and SERT downregulation leading to gut dysmotility. The expression of TRPV1, another ionic channel, has been shown to be increased in the recovery phase only in the colonic mucosa and linked to the persistent visceral hypersensitivity (167). However, other studies have shown a quick restoration of the original phenotype without colonic hypersensitivity to mechanical stimuli (152).

Trinitrobenzene sulfonic acid (TNBS), leads to a Th1 immune response with ulcers in rodents within the first 3 days after instillation. Two weeks after instillation, the inflammation is resolved, but a visceral hypersensitivity, motility dysfunction due to a persistent long-term smooth muscle hyperactivity to acetylcholine, an increased mast cell infiltration in the mucosa, an upregulation of the NMDA-NR1, as well as galanin and tachykinin expression in the mucosa and myenteric plexus, a barrier hyperpermeability and a decreased secretory function through a cyclooxygenase-2 (COX-2) dependent mechanism can be demonstrated (168). The long-lasting symptoms, such as visceral hypersensitivity, are present up to 17 weeks after the induction and involve overexpression of the NMDA receptor NR1 in the spinal cord as well as changes in the distribution and the sensitivity of the colonic afferents. A TRPV1 antagonist, a guanylate cyclase agonist, melatonin and a probiotic (*Bifidobacterium infantis* 35624) were able to rescue this phenotype (169). Mast cells, through their main mediator histamine and its receptors H4R and H1A, play a substantial role in post-inflammatory visceral hypersensitivity (170). Recently, Winston et al. demonstrated a gastric hypersensitivity in 8 weeks-old rats previously exposed to TNBS (171) which positioned this model as a general model for FGID and a good model for patients with FD and IBS overlap symptoms, which is common in clinical practice (172).

Acid acetic, Zymosan and mustard oil are irritants administered directly into the colon of adult or neonatal animals by enema. When administered in pups, long-lasting visceral hypersensitivity has been reported for 8 to 10 weeks for Zymosan and mustard oil and up to 12 weeks for acid acetic (168) without histological damage in adult rats (173). Seven days after acid acetic induction in adult rats, when the inflammatory phase has subsided, a defect of the intestinal barrier function was reported with altered occludin and ZO-1 protein expression in a miR-144-dependent manner (174). The same barrier defect was also observed in rats submitted to the neonatal protocol. When administered directly into the submucosal layer of the stomach using 15 to 20 injections in adult rats, acid acetic promoted gastric hypersensitivity (175). Zymosan and mustard oil sensitized the mechanoreceptor and other neurons present in the colonic wall which persisted after the inflammatory phase (176, 177). Both Zymosan and mustard oil, induced neuronal changes in the spinal cord and the brain by increasing the neuronal excitability as shown by an increased presence of c-FOS positive neurons (178). The latter was associated with an altered expression pattern in the NMDA receptors in females which might be responsible for the female predominance in visceral sensitization following the mustard oil model (179). The stimulating effect of mustard oil on GI motility has been documented in both the upper and lower GI tract (180). Although the increased neuronal activation in the central nervous system in those models would suggest behavioral changes, anxiety-like symptoms have only been described in the zymosan model in which these behavioral symptoms were present during the inflammatory phase and remained present for 2 weeks after the induction, associated with increased c-FOS expression in different brain regions such as the amygdala, prefrontal cortex, periaqueductal gray. and increased TNF-α levels in the colonic mucosa (181).

Iodoacetamide is an alkylating agent administered by gavage in neonate rodents to induce a mild inflammation of the gastric mucosa that is associated with acute changes in sensory and motor function. Inhibition of the glucocorticoid receptors, adrenergic receptors, BDNF or the nerve growth factor (NGF) during the neonatal period suppressed the induced gastric hypersensitivity (171). The inflammation phase in pups is characterized by a thickening of the neuromuscular layer without increased MPO levels. During adulthood, histology and inflammation levels are comparable to control animals while the gastric sensory and motor dysfunction remained present up to 8 weeks after the treatment (182).

### Post-infectious Models

Within the GI tract, the host and billions of micro-organisms are co-existing, creating a unique symbiosis. Products secreted by the microflora influence the gut function by their effect on neurotransmitters, epithelial function, secretion, or muscle contraction (183). The composition of the gut microflora depends on different factors such as diet, geographic position, genetics, and gender. The balance is strongly influenced by changes in the diet, travel or bacterial and parasitic gastrointestinal infections. The occurrence of FGID symptoms after an infectious episode has been found in a range of 3 to 36% of an infected population (156, 184, 185). The latter has been found to alter gut physiology through different mechanisms including the triggering of an inflammatory reaction, alterations at the neurochemical level and immune function, and alterations of the nerve distribution (186). Some of the parasites and bacteria that can infect the human GI tract, can also infect rodents and trigger symptoms or GI abnormalities reminiscent of human FGID.

*Trichinella spiralis* is an intestinal parasite found in humans, rodents, and pigs and is used as a model of post-infectious IBS in mice. For this purpose, mice are infected with 200 to 300 larvae in one gavage. During the acute phase, parasites are evacuated from the organism triggering a Th2 inflammatory phase in both the mucosa and muscular layers (187, 188). The post-infectious phase is defined at 4 weeks post-infection when the inflammatory phase has subsided (188). However, Akiho et al. found that transforming growth factor (TGF)-β remained overexpressed during this post-inflammatory phase and the smooth muscle cells were still hyperreactive to an immune challenge (primary culture of smooth muscle cells incubated with Th2 cytokines TGF-β1 and COX2) in a COX-2 dependent manner (187). Moreover, the long-lasting effects of the infection, such as visceral hyperalgesia which has been reported up to 70 days after infection, were inhibited by selective and non-selective COX-2 inhibitors (187, 189). Data on the effect of COX-2 inhibitors in humans are still lacking. In the post-infectious phase, the small intestine smooth muscle contractility, as well as the mucosal transport, remained altered. The latter shifted from a predominantly cholinergic in normal conditions to a non-cholinergic regulation (188).

Other animal models used infections with *Nippostrongylus brasiliensis* and *Cryptosporidium parvum*, which are both characterized by mast cell hyperplasia, visceral hypersensitivity, motility dysfunction with an increased motor response to excitatory agonists due to a remodeling of the nerve pattern as found in PI-IBS patients long after the infection. Other bacteria have been used including *Campylobacter rodentium, Campylobacter jejuni* or *Salmonella enterica*. However, those infections are less well-characterized regarding their FGID features or have a low success rate in rodents (168).

Overgrowth of *Escherichia coli* in the ileum of IBS and IBD patients has been associated with the expression of the human bacterial colonizing receptor CEACAM6 (190). Expression of the human CEACAM6 in the murine GI tract induced colonization and growth of pathogenic Escherichia coli upon gavage and led to an infection. In this model, mice are treated with the bacteria for 3 days, leading to a transient inflammation, intestinal hyperpermeability, and colonic hypersensitivity. The latter was present until 3 weeks after infection and was associated with a remaining low-grade inflammation and overexpression of the purinergic receptors P2X receptors in the colon (191).

## Food-Related Models of FGID

Food indigestion, intolerance or allergy are major triggers for abdominal symptoms. Due to genetic predisposition, infection or stress, oral tolerance, which is critical in avoiding immune reactions against food antigens, may be disrupted, leading to FGID symptoms. In IBS patients, the ingestion of certain food compounds triggers FGID symptoms (192) and elimination diet strategies, such as low FODMAP or gluten-free diets, are effective in some patients (193).

Food allergy represents a break in the oral tolerance and the consumption of allergens trigger a Th2 response and the activation of mast cells through the IgE pathway. FGID manifestations such as low-grade inflammation, visceral hypersensitivity, increased permeability, have been reported in rodent models of food allergy (194, 195). In those models as well as in humans, gender differences have been described. However, studies are diverging on the effect of the gender with female rodents being more affected than males while in children the prevalence is higher in males. Those differences might be related to the difference in the immune system response to allergens which is strongly influenced by sex hormones (102, 195). Those models differ by the allergen used, which can be egg proteins, peanut components, milk or seafood extracts, but all follow the same pattern with a phase of allergy-induction and a re-challenge with the same allergen several days or weeks after the induction (195). Many of the validation criteria for a rodent allergy model are comparable to the evaluation of the FGID related changes, e.g., presence of histological changes with local and systemic inflammation and activation of eosinophils and mast cells (195). In an elegant study, Aguilera-Lizarraga and Florens demonstrated that the establishment of oral tolerance can be impaired due to stress or intestinal infection, two major triggers of FGID symptoms, without triggering a systemic allergic reaction. Their results showed that this impaired tolerance triggered a mast cell activation through local (but not systemic) IgE, leading to colonic hypersensitivity and hyperpermeability (196, 197).

Obesity is one of the main health problems of our society and affects an increasing number of people over the world. Obesity is often associated with metabolic disorders such as diabetes or hypertension and also with FGID (198). IBS is three times more frequent in obese patients compared to the general population (199) and patients report both upper and lower GI complaints (200). Moreover, studies have reported an increased incidence of GI symptoms among fast-food and western-diet consumers (201, 202). Although, no animal model of FGID includes eating habits or genetic background leading to obesity, several observations in obesity models have pointed out a chronic GI low-grade inflammation and hyperpermeability induced by high fat, high sugar diet (HFHS) (203) which is associated with changes in the microbiota composition (204). Therapeutic strategies targeting the microbiota (205) as well as dietary strategies (204, 206) have shown some promising results in this model on the immune dysfunction and colonic hyperpermeability. Although the effect of obesity on low-grade inflammation, neuropathy, and hyperpermeability is not specific to the gut—some studies reported epigenetic changes in several organs (207)—it will be interesting to further investigate the effect of obesity on the gastro-intestinal features of FGID and on how it affects the outcome and/or the development of FGID.

The imbalance between some bacterial phyla such as Firmicutes/Bacteroidetes has been reported in IBS patients (208). The firmicutes are the predominant butyrate and other short-chain fatty acids (SCFA)-synthesizing bacteria within the colon. Although the benefits of SCFA has been demonstrated, rectal butyrate instillation in animals has been linked to visceral hypersensitivity (209, 210). This hypersensitivity without inflammation involved the enteric glial cells-derived NGF pathway which sensitized the nerve fibers within the colonic wall (211).

## Spontaneous Models of FGID

Spontaneous animal models sharing key characteristics with human FGID are of great value to unravel the complex chain of events ultimately leading to symptoms and to aid in preclinical drug development. Only a few spontaneous models for FGID have been described (212–214) often sharing common features for FD and IBS, mostly in rats.

The BioBreeding rat (BB-rat) is an inbred colony originating from Wistar rats and have been selected for their ability to spontaneously develop type 1 diabetes (215). The BB-rat consists of a diabetes-resistant (control) and a diabetes-prone (BB-DP) strain of which about 50 to 90% develops hyperglycemia depending on their environment (216). Originally mostly used as a model for type 1 diabetes, several groups have demonstrated GI alterations closely mimicking FGID. Indeed, Neu et al. have described changes in intestinal morphology and intestinal permeability before the onset of diabetes (217). In the last 5 years, our lab has described a gastrointestinal phenotype in those BB-DP animals which did not develop diabetes, at all levels of the GI tract. The intestinal changes closely resemble the alterations found in patients with FD and IBS (213, 218–220). Based on these observations, we proposed the normoglycemic BB-DP rat as a spontaneous animal model for FGID. A natural history study of the small intestine demonstrated that the earliest abnormality was an increased intestinal permeability at 50 days of age, followed by an immune cell infiltration, progressing from the mucosa to the myenteric plexus in animals from 110 days onwards (213). This myenteric plexitis is associated with a loss of nitrergic neurons and disturbed motility (213, 220). Concomitantly, an impaired gastric accommodation, like in functional dyspepsia, has been observed in young normoglycemic rats (220). The immune infiltration is affecting the stomach, the small intestine, and the colon and is characterized by activated mast cells and eosinophils (221). Although the barrier defect precedes the infiltration of the immune cells in the small intestine, we observed that in the colon, the infiltration of the immune cells, which is present at the same age as in the jejunum, precedes the hyperpermeability suggesting a different mechanism in both locations. At both levels, we found a positive correlation between mast-cells density and mucosal permeability. Following this immune activation, we also demonstrated colonic hypersensitivity to colorectal distention and anxiety-like behavior in older BB-DP rats, which, however, was not associated with the increased permeability or immune infiltration in the colon (219). Altogether, the BB-rat model is a valid spontaneous animal model for FGID, recapitulating the permeability defect, eosinophil and mast cell predominant immune activation, motility disturbances at different levels of the GI tract, visceral hypersensitivity and behavioral alterations, similar to human FGID.

The Wistar Kyoto (WKY) rat, originally used as a control normotensive strain for the spontaneously hypertensive rats (SHR), has been studied in the last decades as a model of brain-gut dysfunction. Those rats display an exaggerated response to chronic stress compared to Sprague Dawley rats (222–224), associated with a higher susceptibility to develop anxiety-like and depression-like symptoms (225). Regional differences in monoamines concentration within the brain may explain their susceptibility to anxiety and depression (226). As described above, stress is a potent trigger for alterations in gut physiology, especially through the central expression of CRH in key structures involved in stress but also in pain and emotion regulation. Wistar Kyoto rats have an increased response to colorectal distension associated with increased neuronal activation in the cortex (227). Specific inhibition of the CRH pathway in the central amygdala and inhibition of central and peripheral 5HT2B inhibits the increased response to colorectal distension (87, 228). Besides the well-described colonic hypersensitivity (87, 228, 229) and impaired intestinal permeability (223, 224), the WKY rats also display gastric alterations such as an impaired gastric accommodation and a higher sensitivity to gastric distension (230). Interestingly, O'Malley et al. have compared the WKY to Sprague Dawley in a maternal separation paradigm and showed that the susceptibility to gastrointestinal dysfunction in stressed Sprague Dawley is comparable to what is found in non-stressed WKY (231).

The Flinders Sensitive Line (FSL) rat originates from selective breeding from Sprague Dawley and has been selected for their resistance to the choline esterase inhibitor, diisopropyl fluorophosphate (232). Used first as a model of cholinergic-adrenergic hypothesis depression (233), they are now more generally used as a model of depression without comorbidity of anxiety and with a female predominance (234). The effect of the microbiota composition has been studied in those rats showing that their microbiota composition is different (235) and might represent a target to improve the depression phenotype (236). However, only a few studies have investigated the gastrointestinal features in those rats. Some characteristics of functional dyspepsia including a delayed gastric emptying but not an impairment of the gastric accommodation have been described (214). Taking into consideration the link between depression and FGID, a more detailed study of the GI features in the FSL rats may bring some new insights for the link between GI symptoms and depression in FGID.

## Interventional Models of FGID

During abdominal surgery, the opening of the skin and the abdominal cavity triggers adrenergic reflexes involving a spinal loop which temporarily blocks GI motility. Considered as an iatrogenic disorder, postoperative ileus (POI) occurs in most patients undergoing abdominal surgery and is characterized by a transiently impaired GI motility. However, when recovery of bowel function is delayed for more than 3–7 days, this disorder is defined as an illness (237). Several rodent models exist to induce POI: briefly, either the abdominal cavity is opened and exposed to room temperature air for 3 h (238) or the intestinal tract is gently manipulated during 5–10 min (239). In both cases, an impaired GI motility affecting the stomach, small intestine, and colon, an intestinal inflammatory response, and hyperpermeability have been reported. The inflammatory response is associated with increased production of TNFα, IL1α, and IL6 in the early stage, followed by an increase of myeloid cell-derived cytokines, e.g., IL1β and CCL2. During this early stage, the inhibitory reflex pathway is activated, inhibiting gut motility. The transit time is delayed from 12 h up to 1 week after the surgery (240). At 24 h after the surgery, small intestine and colonic transit were delayed and associated with increased cytokine expression within the smooth muscle layer. The role of mast cells in the POI is still controversial as some studies found a mast cell-dependent mechanism in the POI-induced intestinal hyperpermeability and bacterial translocation (241) while other studies found no involvement of these cells by using another type of mast cell knockout (KO) mice (242). While intestinal permeability to bacteria is increased in this model, the TLR2/4 pathway does not seem to be involved (243). Although the role of mast cells is still unsure, the infiltration of resident macrophages through their expression of the alpha7 nicotinic acetylcholine receptors plays a critical role (244). The early cytokines released from the drop of temperature and dry stimulation due to the opening of the abdominal cavity, are potent activators of the macrophages. Furthermore, the afferent nerves activated by the manipulation of the intestine may also activate the resident macrophages and trigger an inflammatory response. In this context, pharmacological or electrical stimulation of the cholinergic anti-inflammatory pathway has been presented as an attractive option to reduce POI (245). Moreover, stimulation of the vagal nerve has been shown to reduce the severity of the POI in animal models (246). As mentioned before, the decrease of the ambient temperature plays an important role in the inflammatory response and also for the activation the thyrotropin-releasing hormone (TRH) in the brain which stimulates gastric motility and secretion for the activation of gastric myenteric cholinergic neurons (247). Several hormones expressed both in the brain and the GI tract are involved in the pathophysiology of POI, including ghrelin, nesfastin-1, somatostatin, TRH, CRF and calcitonin gene-related peptide (CGRP). In the POI model, lower ghrelin concentrations were observed which—like CRF and CGRP—leads to a delayed gastric emptying (248–250). Pharmacological inhibition of somatostatin, a hormonal modulator, in the POI model induced elevated ghrelin levels (251). Centrally, POI activates brain nuclei (supraoptic nucleus, locus coeruleus, paraventricular nucleus of the hypothalamus & rostral raphe pallidus) expressing the nucleobindin2/nesfatin-1 complex which contribute to the decrease in food intake and intestinal transit (252).

Manipulations of the central nervous system have also been described as potential models of GI disorders associated with anxiety-like symptoms. As described before, the limbic system and especially the amygdala are strongly involved in stress-induced colonic hypersensitivity associated with anxiety-like symptoms and the manipulation of this brain region is sufficient to induce colonic pain (253). Direct delivery of corticosterone through a surgically implanted cannula in the central nucleus of the amygdala (CeA) induces a persistent colonic hypersensitivity, which is dependent on CRH, mineralocorticoid and glucocorticoid receptors (169). The activation with a specific agonist of one of those receptors in the CeA, has the same effect on colonic sensitivity as stress (254, 255). Furthermore, the infusion of corticosterone directly into the amygdala leads to epigenetic modifications that enhance the expression of those receptors in a long-term and transmissible manner (256).

## Miscellaneous Models of FGID

Genetic models of FGID include specific KO animals for several receptors, ion channels, and cellular pathways. These models have provided important knowledge of FGID pathophysiology. Mostly used to better understand the pathophysiology of colonic pain, they have demonstrated the importance of BDNF, guanylate cyclase, serotonin transporters, and ion channels. The latter have been extensively described in the literature to be involved in the intestinal mechanoreception and inflammation (257, 258). Therefore, all compounds capable of activation/inhibition of those channels can trigger IBS-like symptoms and especially colonic pain (169), the full description of which is beyond the scope of the current review. Studies in mice deficient for the protease-activated receptor 2 (PAR2) highlighted the importance of this receptor in colonic sensitivity (259), and immune response, notably against *Trichinella spiralis* (260). Other key components of innate immunity, e.g., the TLRs, such as TLR4, are expressed in the intestinal tract and the CNS (261). The activation of TLR4 leads to the activation of inflammatory cascade but also pain behavior through its expression in the spinal cord. Furthermore, TLR4 has been found to be upregulated in the GI tract of patients with IBS (262, 263). Studies in TLR4 KO mice demonstrated a role of the central expression of TLR4 in visceral hypersensitivity following maternal separation stress (264). More generally, in the CRH-induced colonic hypersensitivity and hyperpermeability, TLR4 is a pivotal factor for CRH-mediated modulation of the immune system (159).

Several studies have demonstrated a cross-sensitization between different abdominal organs. In the spinal cord and the brain, the convergence of the sensory neuronal pathways of the different organs is one of the mechanisms underlying this sensory visceral crosstalk (265). Furthermore, within the abdominal cavity, all organs are linked to each other through physical contact and blood circulation. The best-documented model of cross-sensitization is the interaction between the bladder and the colon, in which inflammation in one of the two will affect the other partner as well. Similar to FGID, bladder pain syndrome and bladder hyperactivity syndrome has a female predominance and is often associated with IBS (266). Animal models of bladder irritation, e.g., triggered by protamine sulfate, display an increased colonic hypersensitivity and permeability (267, 268).

## Non-Rodent Models

Although most of the research focuses on rodent models, some other species have been used to investigate the FGID pathophysiology. The guinea pig is a good model to study intestinal motility and the enteric nervous system. The models used are similar to what we previously described in rodents, with the use of stress models such as water avoidance and CRH injection (269, 270). As a model of altered GI transit, several chemical approaches have been used in guinea pigs such as the gavage with mustard oil and serotonin or the injection of TRH. Mustard oil, given orally, induces elongation of the transit time in the upper GI (esophagus) and a decreased transit time in the lower GI part (colon) (271). Ricinoleic acid-induced defecation in the guinea pig is suppressed by a specific tachykinin receptor NK2 antagonist (272).

In rabbits, intracolonic infusion of Zymosan leads to colonic hypersensitivity (272) which is reduced by a tachykinin NK2 receptor antagonist.

Pigs have a comparable GI system to humans, with an equivalent size, anatomy, development and diet preference, which are evidently very different in rodents (273). Also, the enteric nervous system phenotype is comparable to the human counterpart with more complex inter-neuronal connections and plexi compared to rodents (274). It has been shown that pigs have a more highly developed CNS with a complex behavior response to psychosocial stimuli (275) and therefore are more suitable as a model for the response of the GI tract to early life stress in humans. In pigs, the weaning itself is considered a very stressful event (both psychological and physical) which promotes an intestinal barrier defect (276). In a model of early weaning, the piglets are separated from their sow 1 week earlier than usual. In this model, adult pigs display a defect in the small intestinal and colon mucosal barrier function with an elevated electrogenic transport activity, chronic diarrhea associated with an enhanced mast cell activation and an upregulation of the enteric cholinergic population (277, 278) Pretreatment of the stressed animals with a CRH antagonist abolished the stress-induced elevated secretory activity and increased intestinal permeability in jejunum and colon (279). *Ex-vivo* experiments demonstrated that CRH increased permeability via a TNF-α dependent mechanism (280). Similar to rodent models and humans, female pigs are more affected than males by this stress paradigm (276). Despite the differences listed above, pig and rodent models reach the same conclusions on the effect of stress on the GI tract, confirming the critical and harmful effect of early life stress across species.

## Summary and Conclusion

Functional gastrointestinal disorders are complex and multifactorial disorders involving a complex interaction between biological, psychological and social variables that none of the current animal models can reproduce perfectly. The strengths and limitations of the varied models are listed in Table 2. The main limitation of those models remains the societal component of the FGID pathophysiology that is extremely difficult to reproduce in animals. Nevertheless, animal models have brought pivotal insights into the pathophysiology of FGID, including the complex interaction between the gut and the central nervous system, and represent essentials tools for identifying novel therapeutic targets and testing of new generations of pharmaceutical and non-pharmaceutical therapies. Over the years, the improved understanding of the FGID pathophysiology has stimulated the conception of new animal models, which are now more complex and include a combination of causes triggering FGID features, which more closely resembles the human condition. However, with the development of those new multidimensional models using a multitude of slightly different protocols—sometimes not explained in detail in the literature—comes a lack of reproducibility hampering further progression. Several guidelines have been created to address this problem and to enhance scientific rigor (281, 282).

**Table 2:** Strengths and limitations for common models of Functional GI disorders.

**Model**	**Strengths**	**Limitations**	**Part of the GI tract studied**
Pre-natal stress	Allows to study epigenetic changes	Individual variation among animals, around 80 to 95% of animals are sensitized by the stress applied	Small bowel and colon
Maternal separation	Reproduces maternal neglect and mistreatment of FGID patients		Colon
Limited bedding	Non-interventional model, avoids experimenter influence		Colon
Odor shock conditioning	Specifically mimics alterations in learning and fear conditioning		colon
Water avoidance	Strong acute models reproducing a strong stressor and mimicking resilience in an uncomfortable situation	Limited construct validity: physical constraint is not a factor commonly encountered in the etiology of FGID in patients	Stomach Ileum (more affected) colon
Partial restraint stress			Stomach and colon
Crowding stress	Models capture the social component in stress-induced FGID	Social organization, and individual reactions to stress are obviously less standardized and more complicated in humans	Small intestine and colon
Social isolation			Colon
Combined stress	Good model to reproduce anetiology commonly found in human	Because of the more complex interaction of stressors and depending on the protocol used, results tend to be more difficult to reproduce	Colon
Post-inflammatory	Reproduces low-grade inflammation often found in FGID e.g., after infection or in IBD in remission	Limited construct validity: interventional models using irritants/chemicals	Local effect depending on the targeted organ: mainly Stomach and colon
Post-infectious	Model for post-infectious FGID which allow a detailed study of dysbiosis involved in FGID	Different infectious agents compared to humans; most models have used parasitic infections which is uncommon in human FGID	Depending on the infection, small bowel or colon
Food allergy	Murine immune response in case of loss of oral tolerance closely resembles the human counterpart	The nutritional pattern differs between rodents and humans; evidence for immune reaction to food is still limited in human FGID	Colon
Spontaneous models	Non-interventional models, good face and construct validity	Sensitivity to environmental factors (food, stressors…) which makes these model more difficult to reproduce	Stomach, small bowel, colon
Postoperative Ileus	Good construct validity. Allows to study of the mechanisms of interventional surgery as a trigger of intestinal alterations	Intervention is highly operator and experimental condition dependent	Small bowel
Manipulation central nervous system	Suitable for mechanistic studies of the involvement of the central nervous system	Limited construct validity: far from human etiology	Mainly colon
Genetic model	Ideal models to study a specific genetic target and its role in FGID	Compensation phenomena; human FGID is not monogenetic	If KO: all levels of the GI tract If conditional KO: organ targeted
Cross sensitization	Understanding of the overlap in neuronal pathways which is common in human FGID	Interventional models using irritants	Depending on the organs targeted (mainly bladder and colon)

*KO, knockout; FGID, functional gastrointestinal disorders; IBD, inflammatory bowel disease*.

Most of the published studies still suffer from two limitations which weaken their translational relevance. First, most of the available studies focus on the lower GI tract (Table 2), while increasing evidence points out the overlap between the different FGIDs. Many of the models described above are characterized as IBS models but might also be suitable as FD models if alterations in the upper GI tract would be investigated. The second limitation is that the large majority of the pre-clinical studies are performed in male animals to avoid the “hormonal fluctuation” problem although FGID are mainly affecting women. Moreover, recent findings about the impact of sex hormones on the immune response suggest that estrogen is an important player in the onset and development of FGID. For each model presented in this review, at least one study performed in females was available, but often studies comparing both genders were lacking. The early life stress models more frequently addressed the impact of gender because of their methodology, since the stress is applied to pups in a stage when the sex is more difficult to determine. Although the field is slowly changing, studies including females are still underrepresented and those taking the hormonal parameters into account are even fewer. In order to improve the construct validity of the animal models capturing the female predominance of human FGID these studies are awaited in the near future.

## Author Contributions

All authors contributed to the first draft of the manuscript, critically revised subsequent drafts, and approved the final version.

## Conflict of Interest

The authors declare that the research was conducted in the absence of any commercial or financial relationships that could be construed as a potential conflict of interest.

## Abbreviations

5HT: serotonin
5HT1A/1B/2A/2B, serotonin receptor type 1A, 1B, 2A: 2B
ACTH: adreno cortico trophic hormone
AMPA: α-amino-3-hydroxy-5-methyl-4-isoxazolepropionic acid
BB-DP: biobreeding diabetes prone
BDNF: brain-derived neurotrophic factor
CCL2: chemokine ligand 2
CEACAM6: CEA cell adhesion molecule 6
CNS: central nervous system
COX-2: cyclo-oxygenase 2
CGRP: calcitonin gene-related peptide
CRF: corticotropin-releasing factor
CRFR: corticotropin-releasing factor receptor
DSS: dextran sodium sulfate
ELS: early life stress
FD: functional dyspepsia
FGID: functional gastrointestinal disorders
FODMAP, Fermentable Oligo-, Di-: Mono-saccharides And Polyols
FSL: Findler sensitive line
GC-C/cGMP: guanylate cyclase C/cyclic guanylin monophosphate
GI: gastro intestinal
GR: Glucocorticoid receptor
HPA: hypothalamus-pituitary-adrenal
HSHF: high fat high sugar
IFN: interferon
IgE: immune globulin E
IL: interleukin
IBS: irritable bowel syndrom
IBD: inflammatory bowel disease
KO: knock out
MS: maternal separation
MPO: myeloperoxidase activity
MUC2: Mucin 2
NGF: nerve growth factor
NMDA: N-methyl-D-aspartate
P2X: purinergic receptor
PAR2: Protease-activated receptor 2
PI: post infectious
PND: post-natal day
POI: post-operative ileus
SCFA: short chain fatty acids
SERT: serotonin transporter
SHR: spontaneously hypertensive rat
SIS: social isolation stress
TFF3: rail fold factor 3
TLR: toll like receptor
TNBS: Trinitrobenzene sulfonic acid
TNF: tumor necrosis factor
TRH: thyrotropin-releasing hormone
TRPV1: transient receptor potentiate vanilloid 1
VNS: vagal nerve stimulation
WAS: water avoidance stress
WKY: wistar kyoto
ZO-1: Zona occludens 1.

## References

[1] Tack J Talley NJ Camilleri M Holtmann G Hu P Malagelada JR Functional gastroduodenal disorders. Gastroenterology. (2006) 130:1466–79. 10.1053/j.gastro.2005.11.059 16678560

[2] Longstreth GF Thompson WG Chey WD Houghton LA Mearin F Spiller RC. Functional bowel disorders. Gastroenterology. (2006) 130:1480–91. 10.1053/j.gastro.2005.11.061 16678561

[3] Drossman DA Functional gastrointestinal disorders: history, pathophysiology, clinical features and Rome IV. Gastroenterology. (2016) 150:1262–79. 10.1053/j.gastro.2016.02.032 27144617

[4] Camilleri M Bueno L Andresen V de Ponti F Choi MG Lembo A Pharmacological, pharmacokinetic, and pharmacogenomic aspects of functional gastrointestinal disorders. Gastroenterology. (2016) 150:1319–31. 10.1053/j.gastro.2016.02.029 27144621

[5] Vanheel H Farre R. Changes in gastrointestinal tract function and structure in functional dyspepsia. Nat Rev Gastroenterol Hepatol. (2013) 10:142–9. 10.1038/nrgastro.2012.255 23318268

[6] Matricon J Meleine M Gelot A Piche T Dapoigny M Muller E. Review article: associations between immune activation, intestinal permeability and the irritable bowel syndrome. Aliment Pharmacol Ther. (2012) 36:1009–31. 10.1111/apt.12080 23066886

[7] vanheel H Vicario M Boesmans W Vanuytsel T Salvo-Romero E Tack J. Activation of eosinophils and mast cells in functional dyspepsia: an ultrastructural evaluation. Sci Rep. (2018) 8:5383. 10.1038/s41598-018-23620-y 29599471 PMC5876347

[8] Robles A Perez Ingles D Myneedu K Deoker A Sarosiek I Zuckerman MJ. Mast cells are increased in the small intestinal mucosa of patients with irritable bowel syndrome: a systematic review and meta-analysis. Neurogastroenterol Motil. (2019) 31:e13718. 10.1111/nmo.13718 31498961

[9] Farre R Vicario M. Abnormal barrier function in gastrointestinal disorders. Handb Exp Pharmacol. (2017) 239:193–217. 10.1007/164_2016_107 27995392

[10] Balemans D Boeckxstaens GE Talavera K Wouters MM. Transient receptor potential ion channel function in sensory transduction and cellular signaling cascades underlying visceral hypersensitivity. Am J Physiol Gastrointest Liver Physiol. (2017) 312:G635–48. 10.1152/ajpgi.00401.2016 28385695

[11] Simren M Tornblom H Palsson OS van Oudenhove L Whitehead WE Tack J Cumulative effects of psychologic distress, visceral hypersensitivity, and abnormal transit on patient-reported outcomes in irritable bowel syndrome. Gastroenterology. (2019) 157:391–402.e2. 10.1053/j.gastro.2019.04.019 31022401

[12] Barbara G Stanghellini V de Giorgio R Corinaldesi R. Functional gastrointestinal disorders and mast cells: implications for therapy. Neurogastroenterol Motil. (2006) 18:6–17. 10.1111/j.1365-2982.2005.00685.x 16371078

[13] Holtmann G Shah A Morrison M Pathophysiology of functional gastrointestinal disorders: a holistic overview. Dig Dis. (2017) 35(Suppl. 1):5–13. 10.1159/000485409 29421808

[14] Beeckmans D Riethorst D Augustijns P Vanuytsel T Farre R Tack J. Altered duodenal bile salt concentration and receptor expression in functional dyspepsia. United Eur Gastroenterol J. (2018) 6:1347–55. 10.1177/2050640618799120 30386607 PMC6206534

[15] Zhong L Shanahan ER Raj A Koloski NA Fletcher L Morrison M. Dyspepsia and the microbiome: time to focus on the small intestine. Gut. (2017) 66:1168–9. 10.1136/gutjnl-2016-312574 27489239

[16] Pittayanon R Lau JT Yuan Y Leontiadis GI Tse F Surette M. Gut microbiota in patients with irritable bowel syndrome-a systematic review. Gastroenterology. (2019) 157:97–108. 10.1053/j.gastro.2019.03.049 30940523

[17] Igarashi M Nakae H Matsuoka T Takahashi S Hisada T Tomita J. Alteration in the gastric microbiota and its restoration by probiotics in patients with functional dyspepsia. BMJ Open Gastroenterol. (2017) 4:e000144. 10.1136/bmjgast-2017-000144 28761692 PMC5508964

[18] Mayer EA Tillisch K. The brain-gut axis in abdominal pain syndromes. Annu Rev Med. (2011) 62:381–96. 10.1146/annurev-med-012309-103958 21090962 PMC3817711

[19] Yi L Sun H Ge C Chen Y Peng H Jiang Y. Role of insular cortex in visceral hypersensitivity model in rats subjected to chronic stress. Psychiatry Res. (2014) 220:1138–43. 10.1016/j.psychres.2014.09.019 25446465

[20] Pinto-Sanchez MI Hall GB Ghajar K Nardelli A Bolino C Lau JT. Probiotic Bifidobacterium longum NCC3001 reduces depression scores and alters brain activity: a pilot study in patients with irritable bowel syndrome. Gastroenterology. (2017) 153:448–59.e8. 10.1053/j.gastro.2017.05.003 28483500

[21] van Oudenhove L Aziz Q. The role of psychosocial factors and psychiatric disorders in functional dyspepsia. Nat Rev Gastroenterol Hepatol. (2013) 10:158–67. 10.1038/nrgastro.2013.10 23358396

[22] van Oudenhove L vandenberghe J Vos R Fischler B Demyttenaere K Tack J. Abuse history, depression, and somatization are associated with gastric sensitivity and gastric emptying in functional dyspepsia. Psychosom Med. (2011) 73:648–55. 10.1097/PSY.0b013e31822f32bf 21949416

[23] Ulrich-Lai YM Herman JP. Neural regulation of endocrine and autonomic stress responses. Nat Rev Neurosci. (2009) 10:397–409. 10.1038/nrn2647 19469025 PMC4240627

[24] Song H Fang F Arnberg FK Mataix-Cols D Fernández de la Cruz L Almqvist C. Stress related disorders and risk of cardiovascular disease: population based, sibling controlled cohort study. BMJ. (2019) 365:l1255. 10.1136/bmj.l1255 30971390 PMC6457109

[25] Kelly RR McDonald LT Jensen NR Sidles SJ LaRue AC. Impacts of psychological stress on osteoporosis: clinical implications and treatment interactions. Front Psychiatry. (2019) 10:200. 10.3389/fpsyt.2019.00200 31024360 PMC6465575

[26] Afrisham R Paknejad M Soliemanifar O Sadegh-Nejadi S Meshkani R Ashtary-Larky D. The influence of psychological stress on the initiation and progression of diabetes and cancer. Int J Endocrinol Metab. (2019) 17:e67400. 10.5812/ijem.67400 31372166 PMC6628619

[27] Yang L Zhao Y Wang Y Liu L Zhang X Li B The effects of psychological stress on depression. Curr Neuropharmacol. (2015) 13:494–504. 10.2174/1570159X1304150831150507 26412069 PMC4790405

[28] Austin KW Ameringer SW Cloud LJ. An integrated review of psychological stress in Parkinson's disease: biological mechanisms and symptom and health outcomes. Parkinsons Dis. (2016) 2016:9869712. 10.1155/2016/9869712 28058129 PMC5183774

[29] Haass-Koffler CL Bartlett SE. Stress and addiction: contribution of the corticotropin releasing factor (CRF) system in neuroplasticity. Front Mol Neurosci. (2012) 5:91. 10.3389/fnmol.2012.00091 22973190 PMC3434418

[30] Tamashiro KL Nguyen MM Sakai RR. Social stress: from rodents to primates. Front Neuroendocrinol. (2005) 26:27–40. 10.1016/j.yfrne.2005.03.001 15862183

[31] Bhatia V Tandon RK. Stress and the gastrointestinal tract. J Gastroenterol Hepatol. (2005) 20:332–9. 10.1111/j.1440-1746.2004.03508.x 15740474

[32] Bunnett NW. The stressed gut: contributions of intestinal stress peptides to inflammation and motility. Proc Natl Acad Sci USA. (2005) 102:7409. 10.1073/pnas.0503092102 15899972 PMC1140465

[33] Smith SM Vale WW. The role of the hypothalamic-pituitary-adrenal axis in neuroendocrine responses to stress. Dialogues Clin Neurosci. (2006) 8:383–95. 17290797 10.31887/DCNS.2006.8.4/ssmith PMC3181830

[34] Munck A Guyre PM Holbrook NJ. Physiological functions of glucocorticoids in stress and their relation to pharmacological actions. Endocr Rev. (1984) 5:25–44. 10.1210/edrv-5-1-25 6368214

[35] McEwen BS. Brain on stress: how the social environment gets under the skin. Proc Natl Acad Sci USA. (2012) 109(Suppl. 2):17180–5. 10.1073/pnas.1121254109 23045648 PMC3477378

[36] Tache Y Perdue MH. Role of peripheral CRF signalling pathways in stress-related alterations of gut motility and mucosal function. Neurogastroenterol Motil. (2004) 16(Suppl. 1):137–42. 10.1111/j.1743-3150.2004.00490.x 15066020

[37] Martinez V Wang L Rivier J Grigoriadis D Tache Y. Central CRF, urocortins and stress increase colonic transit via CRF1 receptors while activation of CRF2 receptors delays gastric transit in mice. J Physiol. (2004) 556:221–34. 10.1113/jphysiol.2003.059659 14755002 PMC1664879

[38] Martinez V Wang L Rivier JE Vale W Tache Y. Differential actions of peripheral corticotropin-releasing factor (CRF), urocortin II, and urocortin III on gastric emptying and colonic transit in mice: role of CRF receptor subtypes 1 and 2. J Pharmacol Exp Ther. (2002) 301:611–7. 10.1124/jpet.301.2.611 11961064

[39] Yin Y Dong L Yin D. Peripheral and central administration of exogenous urocortin 1 disrupts the fasted motility pattern of the small intestine in rats via the corticotrophin releasing factor receptor 2 and a cholinergic mechanism. J Gastroenterol Hepatol. (2008) 23:e79–87. 10.1111/j.1440-1746.2007.05142.x 17944898

[40] Larauche M Gourcerol G Wang L Pambukchian K Brunnhuber S Adelson DW. Cortagine, a CRF1 agonist, induces stresslike alterations of colonic function and visceral hypersensitivity in rodents primarily through peripheral pathways. Am J Physiol Gastrointest Liver Physiol. (2009) 297:G215–27. 10.1152/ajpgi.00072.2009 19407218 PMC2711753

[41] Trimble N Johnson AC Foster A Greenwood-van Meerveld B. Corticotropin-releasing factor receptor 1-deficient mice show decreased anxiety and colonic sensitivity. Neurogastroenterol Motil. (2007) 19:754–60. 10.1111/j.1365-2982.2007.00951.x 17539891

[42] Million M Wang L Wang Y Adelson DW Yuan PQ Maillot C. CRF2 receptor activation prevents colorectal distension induced visceral pain and spinal ERK1/2 phosphorylation in rats. Gut. (2006) 55:172–81. 10.1136/gut.2004.051391 15985561 PMC1856510

[43] la Fleur SE Wick EC Idumalla PS Grady EF Bhargava A. Role of peripheral corticotropin-releasing factor and urocortin II in intestinal inflammation and motility in terminal ileum. Proc Natl Acad Sci USA. (2005) 102:7647–52. 10.1073/pnas.0408531102 15883387 PMC1140406

[44] Barreau F Cartier C Ferrier L Fioramonti J Bueno L. Nerve growth factor mediates alterations of colonic sensitivity and mucosal barrier induced by neonatal stress in rats. Gastroenterology. (2004) 127:524–34. 10.1053/j.gastro.2004.05.019 15300585

[45] Hagiwara SI Kaushal E Paruthiyil S Pasricha PJ Hasdemir B Bhargava A. Gastric corticotropin-releasing factor influences mast cell infiltration in a rat model of functional dyspepsia. PLoS ONE. (2018) 13:e0203704. 10.1371/journal.pone.0203704 30192883 PMC6128656

[46] Nicholl BI Halder SL Macfarlane GJ Thompson DG O'Brien S Musleh M. Psychosocial risk markers for new onset irritable bowel syndrome–results of a large prospective population-based study. Pain. (2008) 137:147–55. 10.1016/j.pain.2007.08.029 17928145 PMC2441776

[47] Vanuytsel T van Wanrooy S Vanheel H Vanormelingen C Verschueren S Houben E. Psychological stress and corticotropin-releasing hormone increase intestinal permeability in humans by a mast cell-dependent mechanism. Gut. (2014) 63:1293–9. 10.1136/gutjnl-2013-305690 24153250

[48] Martin TD Chan SS Hart AR. Environmental factors in the relapse and recurrence of inflammatory bowel disease: a review of the literature. Dig Dis Sci. (2015) 60:1396–405. 10.1007/s10620-014-3437-3 25407806

[49] O'Mahony SM Clarke G Dinan TG Cryan JF. Early-life adversity and brain development: is the microbiome a missing piece of the puzzle? Neuroscience. (2017) 342:37–54. 10.1016/j.neuroscience.2015.09.068 26432952

[50] Mayer EA Naliboff BD Chang L Coutinho SV. Stress and irritable bowel syndrome. Am J Physiol Gastrointest Liver Physiol. (2001) 280:G519–24. 10.1152/ajpgi.2001.280.4.G519 11254476

[51] Klooker TK Braak B Painter RC de Rooij SR van Elburg RM van den Wijngaard RM. Exposure to severe wartime conditions in early life is associated with an increased risk of irritable bowel syndrome: a population-based cohort study. Am J Gastroenterol. (2009) 104:2250–6. 10.1038/ajg.2009.282 19513027

[52] Camp LL Rudy JW. Changes in the categorization of appetitive and aversive events during postnatal development of the rat. Dev Psychobiol. (1988) 21:25–42. 10.1002/dev.420210103 3338626

[53] Moriceau S Sullivan RM. Corticosterone influences on Mammalian neonatal sensitive-period learning. Behav Neurosci. (2004) 118:274–81. 10.1037/0735-7044.118.2.274 15113251 PMC1868531

[54] Macri S Neonatal corticosterone administration in rodents as a tool to investigate the maternal programming of emotional and immune domains. Neurobiol Stress. (2017) 6:22–30. 10.1016/j.ynstr.2016.12.001 28229106 PMC5314439

[55] Walker CD Perrin M Vale W Rivier C. Ontogeny of the stress response in the rat: role of the pituitary and the hypothalamus. Endocrinology. (1986) 118:1445–51. 10.1210/endo-118-4-1445 3004915

[56] Levine S Glick D Nakane PK. Adrenal and plasma corticosterone and vitamin A in rat adrenal glands during postnatal development. Endocrinology. (1967) 80:910–4. 10.1210/endo-80-5-910 4290303

[57] Sapolsky RM Meaney MJ. Maturation of the adrenocortical stress response: neuroendocrine control mechanisms and the stress hyporesponsive period. Brain Res. (1986) 396:64–76. 10.1016/0165-0173(86)90010-X 3011218

[58] Levine S. Primary social relationships influence the development of the hypothalamic–pituitary–adrenal axis in the rat. Physiol Behav. (2001) 73:255–60. 10.1016/S0031-9384(01)00496-6 11438350

[59] Suchecki D Mozaffarian D Gross G Rosenfeld P Levine S. Effects of maternal deprivation on the ACTH stress response in the infant rat. Neuroendocrinology. (1993) 57:204–12. 10.1159/000126361 8389994

[60] Vazquez DM Lopez JF van Hoers H Watson SJ Levine S. Maternal deprivation regulates serotonin 1A and 2A receptors in the infant rat. Brain Res. (2000) 855:76–82. 10.1016/S0006-8993(99)02307-0 10650132

[61] Muret L Priou A Oliver C Grino M Stimulation of adrenocorticotropin secretion by insulin-induced hypoglycemia in the developing rat involves arginine vasopressin but not corticotropin-releasing factor. Endocrinology. (1992) 130:2725–32. 10.1210/endo.130.5.1315256 1315256

[62] Zelena D Domokos A Barna I Mergl Z Haller J Makara GB. Control of the hypothalamo-pituitary-adrenal axis in the neonatal period: adrenocorticotropin and corticosterone stress responses dissociate in vasopressin-deficient brattleboro rats. Endocrinology. (2008) 149:2576–83. 10.1210/en.2007-1537 18276753

[63] Opendak M Robinson-Drummer P Blomkvist A Zanca RM Wood K Jacobs L. Neurobiology of maternal regulation of infant fear: the role of mesolimbic dopamine and its disruption by maltreatment. Neuropsychopharmacology. (2019) 44:1247–57. 10.1038/s41386-019-0340-9 30758321 PMC6784970

[64] Claflin DI Schmidt KD Vallandingham ZD Kraszpulski M Hennessy MB. Influence of postnatal glucocorticoids on hippocampal-dependent learning varies with elevation patterns and administration methods. Neurobiol Learn Mem. (2017) 143:77–87. 10.1016/j.nlm.2017.05.010 28545908 PMC5540754

[65] Daun KA Fuchigami T Koyama N Maruta N Ikenaka K Hitoshi S. Early maternal and social deprivation expands neural stem cell population size and reduces hippocampus/amygdala-dependent fear memory. Front Neurosci. (2020) 14:22. 10.3389/fnins.2020.00022 32063832 PMC7000530

[66] Winston JH Li Q Sarna SK. Chronic prenatal stress epigenetically modifies spinal cord BDNF expression to induce sex-specific visceral hypersensitivity in offspring. Neurogastroenterol Motil. (2014) 26:715–30. 10.1111/nmo.12326 24588943 PMC3997587

[67] Jasarevic E Howerton CL Howard CD Bale TL. Alterations in the vaginal microbiome by maternal stress are associated with metabolic reprogramming of the offspring gut and brain. Endocrinology. (2015) 156:3265–76. 10.1210/en.2015-1177 26079804 PMC4541625

[68] Golubeva AV Crampton S Desbonnet L Edge D O'Sullivan O Lomasney KW. Prenatal stress-induced alterations in major physiological systems correlate with gut microbiota composition in adulthood. Psychoneuroendocrinology. (2015) 60:58–74. 10.1016/j.psyneuen.2015.06.002 26135201

[69] Molet J Maras PM Avishai-Eliner S Baram TZ. Naturalistic rodent models of chronic early-life stress. Dev Psychobiol. (2014) 56:1675–88. 10.1002/dev.21230 24910169 PMC4777289

[70] Orso R Wearick-Silva LE Creutzberg KC Centeno-Silva A Glusman Roithmann L Pazzin R. Maternal behavior of the mouse dam toward pups: implications for maternal separation model of early life stress. Stress. (2018) 21:19–27. 10.1080/10253890.2017.1389883 29041860

[71] Plotsky PM Meaney MJ. Early, postnatal experience alters hypothalamic corticotropin-releasing factor (CRF) mRNA, median eminence CRF content and stress-induced release in adult rats. Brain Res Mol Brain Res. (1993) 18:195–200. 10.1016/0169-328X(93)90189-V 8497182

[72] McCormick CM Kehoe P Kovacs S. Corticosterone release in response to repeated, short episodes of neonatal isolation: evidence of sensitization. Int J Dev Neurosci. (1998) 16:175–85. 10.1016/S0736-5748(98)00026-4 9785114

[73] Plotsky PM Thrivikraman KV Nemeroff CB Caldji C Sharma S Meaney MJ. Long-term consequences of neonatal rearing on central corticotropin-releasing factor systems in adult male rat offspring. Neuropsychopharmacology. (2005) 30:2192–204. 10.1038/sj.npp.1300769 15920504

[74] Moussaoui N Jacobs JP Larauche M Biraud M Million M Mayer E. Chronic early-life stress in rat pups alters basal corticosterone, intestinal permeability, and fecal microbiota at weaning: influence of sex. J Neurogastroenterol Motil. (2017) 23:135–43. 10.5056/jnm16105 27829577 PMC5216644

[75] Li B Lee C Zani A Zani-Ruttenstock E Ip W Chi L. Early maternal separation induces alterations of colonic epithelial permeability and morphology. Pediatr Surg Int. (2014) 30:1217–22. 10.1007/s00383-014-3611-x 25358892

[76] Meleine M Boudieu L Gelot A Muller E Lashermes A Matricon J. Comparative effects of alpha2delta-1 ligands in mouse models of colonic hypersensitivity. World J Gastroenterol. (2016) 22:7111–23. 10.3748/wjg.v22.i31.7111 27610021 PMC4988313

[77] van den Wijngaard RM Stanisor OI van Diest SA Welting O Wouters MM Cailotto C. Susceptibility to stress induced visceral hypersensitivity in maternally separated rats is transferred across generations. Neurogastroenterol Motil. (2013) 25:e780–90. 10.1111/nmo.12202 23965154

[78] van Den Wijngaard RM Klooker TK Welting O Stanisor OI Wouters MM van Der Coelen D. Essential role for TRPV1 in stress-induced (mast cell-dependent) colonic hypersensitivity in maternally separated rats. Neurogastroenterol Motil. (2009) 21:1107–94. 10.1111/j.1365-2982.2009.01339.x 19523146

[79] O'Malley D Liston M Hyland NP Dinan TG Cryan JF. Colonic soluble mediators from the maternal separation model of irritable bowel syndrome activate submucosal neurons via an interleukin-6-dependent mechanism. Am J Physiol Gastrointest Liver Physiol. (2011) 300:G241–52. 10.1152/ajpgi.00385.2010 21109592

[80] Barreau F Ferrier L Fioramonti J Bueno L. Neonatal maternal deprivation triggers long term alterations in colonic epithelial barrier and mucosal immunity in rats. Gut. (2004) 53:501–6. 10.1136/gut.2003.024174 15016743 PMC1774003

[81] Gareau MG Jury J Perdue MH. Neonatal maternal separation of rat pups results in abnormal cholinergic regulation of epithelial permeability. Am J Physiol Gastrointest Liver Physiol. (2007) 293:G198–203. 10.1152/ajpgi.00392.2006 17510196

[82] Li B Lee C Filler T Hock A Wu RY Li Q. Inhibition of corticotropin-releasing hormone receptor 1 and activation of receptor 2 protect against colonic injury and promote epithelium repair. Sci Rep. (2017) 7:46616. 10.1038/srep46616 28492284 PMC5425914

[83] Ackerman SH Hofer MA Weiner H. Predisposition to gastric erosions in the rat: behavioral and nutritional effects of early maternal separation. Gastroenterology. (1978) 75:649–54. 10.1016/S0016-5085(19)31674-9 710833

[84] Tominaga K Fujikawa Y Tanaka F Kamata N Yamagami H Tanigawa T. Structural changes in gastric glial cells and delayed gastric emptying as responses to early life stress and acute adulthood stress in rats. Life Sci. (2016) 148:254–9. 10.1016/j.lfs.2016.02.025 26874028

[85] Biagini G Pich EM Carani C Marrama P Agnati LF. Postnatal maternal separation during the stress hyporesponsive period enhances the adrenocortical response to novelty in adult rats by affecting feedback regulation in the CA1 hippocampal field. Int J Dev Neurosci. (1998) 16:187–97. 10.1016/S0736-5748(98)00019-7 9785115

[86] Daniels WM Pietersen CY Carstens ME Stein DJ. Maternal separation in rats leads to anxiety-like behavior and a blunted ACTH response and altered neurotransmitter levels in response to a subsequent stressor. Metab Brain Dis. (2004) 19:3–14. 10.1023/B:MEBR.0000027412.19664.b3 15214501

[87] O'Mahony SM Bulmer DC Coelho AM Fitzgerald P Bongiovanni C Lee K. 5-HT(2B) receptors modulate visceral hypersensitivity in a stress-sensitive animal model of brain-gut axis dysfunction. Neurogastroenterol Motil. (2010). 22:573–8.e124. 10.1111/j.1365-2982.2009.01432.x 20003079

[88] Martisova E Solas M Horrillo I Ortega JE Meana JJ Tordera RM. Long lasting effects of early-life stress on glutamatergic/GABAergic circuitry in the rat hippocampus. Neuropharmacology. (2012) 62:1944–53. 10.1016/j.neuropharm.2011.12.019 22245561

[89] Matthews K Dalley JW Matthews C Tsai TH Robbins TW. Periodic maternal separation of neonatal rats produces region- and gender-specific effects on biogenic amine content in postmortem adult brain. Synapse. (2001) 40:1–10. 10.1002/1098-2396(200104)40:1<1::AID-SYN1020>3.0.CO;2-E 11170216

[90] Yu Y-C Li J Zhang M Pan J-C Yu Y Zhang J-B. Resveratrol improves brain-gut axis by regulation of 5-HT-dependent signaling in the rat model of irritable bowel syndrome. Front Cell Neurosci. (2019) 13:30. 10.3389/fncel.2019.00030 30800058 PMC6375832

[91] Ford AC Talley NJ Schoenfeld PS Quigley EM Moayyedi P. Efficacy of antidepressants and psychological therapies in irritable bowel syndrome: systematic review and meta-analysis. Gut. (2009) 58:367–78. 10.1136/gut.2008.163162 19001059

[92] Bian ZX Zhang M Han QB Xu HX Sung JJ. Analgesic effects of JCM-16021 on neonatal maternal separation-induced visceral pain in rats. World J Gastroenterol. (2010) 16:837–45. 10.3748/wjg.v16.i7.837 20143462 PMC2825330

[93] Arborelius L Eklund MB. Both long and brief maternal separation produces persistent changes in tissue levels of brain monoamines in middle-aged female rats. Neuroscience. (2007) 145:738–50. 10.1016/j.neuroscience.2006.12.007 17222517

[94] Manabe N Tanaka T Hata J Kusunoki H Haruma K. Pathophysiology underlying irritable bowel syndrome–from the viewpoint of dysfunction of autonomic nervous system activity. J Smooth Muscle Res. (2009) 45:15–23. 10.1540/jsmr.45.15 19377269

[95] Pickering C Gustafsson L Cebere A Nylander I Liljequist S Repeated maternal separation of male Wistar rats alters glutamate receptor expression in the hippocampus but not the prefrontal cortex. Brain Res. (2006) 1099:101–8. 10.1016/j.brainres.2006.04.136 16784730

[96] Ryan B Musazzi L Mallei A Tardito D Gruber SH El Khoury A. Remodelling by early-life stress of NMDA receptor-dependent synaptic plasticity in a gene-environment rat model of depression. Int J Neuropsychopharmacol. (2009) 12:553–9. 10.1017/S1461145708009607 18976544

[97] Lajud N Roque A Cajero M Gutierrez-Ospina G Torner L. Periodic maternal separation decreases hippocampal neurogenesis without affecting basal corticosterone during the stress hyporesponsive period, but alters HPA axis and coping behavior in adulthood. Psychoneuroendocrinology. (2012) 37:410–20. 10.1016/j.psyneuen.2011.07.011 21862224

[98] Baram TZ Davis EP Obenaus A Sandman CA Small SL Solodkin A. Fragmentation and unpredictability of early-life experience in mental disorders. Am J Psychiatry. (2012) 169:907–15. 10.1176/appi.ajp.2012.11091347 22885631 PMC3483144

[99] Gilles EE Schultz L Baram TZ. Abnormal corticosterone regulation in an immature rat model of continuous chronic stress. Pediatr Neurol. (1996) 15:114–9. 10.1016/0887-8994(96)00153-1 8888044 PMC3415889

[100] Walker CD Bath KG Joels M Korosi A Larauche M Lucassen PJ. Chronic early life stress induced by limited bedding and nesting (LBN) material in rodents: critical considerations of methodology, outcomes and translational potential. Stress. (2017) 20:421–48. 10.1080/10253890.2017.1343296 28617197 PMC5705407

[101] Ivy AS Brunson KL Sandman C Baram TZ. Dysfunctional nurturing behavior in rat dams with limited access to nesting material: a clinically relevant model for early-life stress. Neuroscience. (2008) 154:1132–42. 10.1016/j.neuroscience.2008.04.019 18501521 PMC2517119

[102] Holschneider DP Guo Y Mayer EA Wang Z. Early life stress elicits visceral hyperalgesia and functional reorganization of pain circuits in adult rats. Neurobiol Stress. (2016) 3:8–22. 10.1016/j.ynstr.2015.12.003 26751119 PMC4700548

[103] Guo Y Wang Z Mayer EA Holschneider DP. Neonatal stress from limited bedding elicits visceral hyperalgesia in adult rats. Neuroreport. (2015) 26:13–6. 10.1097/WNR.0000000000000292 25426824 PMC4246414

[104] Prusator DK Greenwood-van Meerveld B. Gender specific effects of neonatal limited nesting on viscerosomatic sensitivity and anxiety-like behavior in adult rats. Neurogastroenterol Motil. (2015) 27:72–81. 10.1111/nmo.12472 25394875

[105] Youssef M Atsak P Cardenas J Kosmidis S Leonardo ED Dranovsky A. Early life stress delays hippocampal development and diminishes the adult stem cell pool in mice. Sci Rep. (2019) 9:4120. 10.1038/s41598-019-40868-0 30858462 PMC6412041

[106] Moriceau S Sullivan RM. Maternal presence serves as a switch between learning fear and attraction in infancy. Nat Neurosci. (2006) 9:1004–6. 10.1038/nn1733 16829957 PMC1560090

[107] Greenwood-van Meerveld B Johnson AC. Stress-induced chronic visceral pain of gastrointestinal origin. Front Syst Neurosci. (2017) 11:86. 10.3389/fnsys.2017.00086 29213232 PMC5702626

[108] Chaloner A Greenwood-van Meerveld B. Sexually dimorphic effects of unpredictable early life adversity on visceral pain behavior in a rodent model. J Pain. (2013) 14:270–80. 10.1016/j.jpain.2012.11.008 23348370

[109] Ligon C Mohammadi E Ge P Hannig G Higgins C Greenwood-Van Meerveld B. Linaclotide inhibits colonic and urinary bladder hypersensitivity in adult female rats following unpredictable neonatal stress. Neurogastroenterol Motil. (2018) 30:e13375. 10.1111/nmo.13375 29797376

[110] Takada M Nishida K Kataoka-Kato A Gondo Y Ishikawa H Suda K. Probiotic Lactobacillus casei strain Shirota relieves stress-associated symptoms by modulating the gut–brain interaction in human and animal models. Neurogastroenterol Motil. (2016) 28:1027–36. 10.1111/nmo.12804 26896291

[111] Lee JY Kim N Nam RH Sohn SH Lee SM Choi D. Probiotics reduce repeated water avoidance stress-induced colonic microinflammation in Wistar rats in a sex-specific manner. PLoS ONE. (2017) 12:e0188992. 10.1371/journal.pone.0188992 29244820 PMC5731730

[112] Keita AV Soderholm JD Ericson AC. Stress-induced barrier disruption of rat follicle-associated epithelium involves corticotropin-releasing hormone, acetylcholine, substance P, and mast cells. Neurogastroenterol Motil. (2010) 22:770–8.e221–2. 10.1111/j.1365-2982.2010.01471.x 20149111

[113] Keita AV Carlsson AH Cigehn M Ericson AC McKay DM Soderholm JD. Vasoactive intestinal polypeptide regulates barrier function via mast cells in human intestinal follicle-associated epithelium and during stress in rats. Neurogastroenterol Motil. (2013) 25:e406–17. 10.1111/nmo.12127 23600853

[114] Nozu T Miyagishi S Nozu R Takakusaki K Okumura T. Repeated water avoidance stress induces visceral hypersensitivity: role of interleukin-1, interleukin-6, and peripheral corticotropin-releasing factor. J Gastroenterol Hepatol. (2017) 32:1958–65. 10.1111/jgh.13787 28299830

[115] Da Silva S Robbe-Masselot C Ait-Belgnaoui A Mancuso A Mercade-Loubiere M Salvador-Cartier C. Stress disrupts intestinal mucus barrier in rats via mucin O-glycosylation shift: prevention by a probiotic treatment. Am J Physiol Gastrointest Liver Physiol. (2014) 307:G420–9. 10.1152/ajpgi.00290.2013 24970779

[116] Zhang L Song J Bai T Qian W Hou XH. Stress induces more serious barrier dysfunction in follicle-associated epithelium than villus epithelium involving mast cells and protease-activated receptor-2. Sci Rep. (2017) 7:4950. 10.1038/s41598-017-05064-y 28694438 PMC5503989

[117] Zhang J Song L Wang Y Liu C Zhang L Zhu S. Beneficial effect of butyrate-producing Lachnospiraceae on stress-induced visceral hypersensitivity in rats. J Gastroenterol Hepatol. (2019) 34:1368–76. 10.1111/jgh.14536 30402954 PMC7379616

[118] Yoshikawa K Kurihara C Furuhashi H Takajo T Maruta K Yasutake Y. Psychological stress exacerbates NSAID-induced small bowel injury by inducing changes in intestinal microbiota and permeability via glucocorticoid receptor signaling. J Gastroenterol. (2017) 52:61–71. 10.1007/s00535-016-1205-1 27075753

[119] Nozu T Kumei S Takakusaki K Okumura T. Water-avoidance stress enhances gastric contractions in freely moving conscious rats: role of peripheral CRF receptors. J Gastroenterol. (2014) 49:799–805. 10.1007/s00535-013-0828-8 23645119

[120] Miwa H Koseki J Oshima T Hattori T Kase Y Kondo T. Impairment of gastric accommodation induced by water-avoidance stress is mediated by 5-HT2B receptors. Neurogastroenterol Motil. (2016) 28:765–78. 10.1111/nmo.12775 26833428

[121] Llorca-Torralba M Suarez-Pereira I Bravo L Camarena-Delgado C Garcia-Partida JA Mico JA. Chemogenetic silencing of the locus coeruleus-basolateral amygdala pathway abolishes pain-induced anxiety and enhanced aversive learning in rats. Biol Psychiatry. (2019) 85:1021–35. 10.1016/j.biopsych.2019.02.018 30987747

[122] Larsson MB Tillisch K Craig AD Engstrom M Labus J Naliboff B. Brain responses to visceral stimuli reflect visceral sensitivity thresholds in patients with irritable bowel syndrome. Gastroenterology. (2012) 142:463–72.e3. 10.1053/j.gastro.2011.11.022 22108191 PMC3288538

[123] Labus JS Hubbard CS Bueller J Ebrat B Tillisch K Chen M. Impaired emotional learning and involvement of the corticotropin-releasing factor signaling system in patients with irritable bowel syndrome. Gastroenterology. (2013) 145:1253–61.e1–3. 10.1053/j.gastro.2013.08.016 23954313 PMC4069031

[124] Kret ME de Gelder B. A review on sex differences in processing emotional signals. Neuropsychologia. (2012) 50:1211–21. 10.1016/j.neuropsychologia.2011.12.022 22245006

[125] Labus JS Gupta A Coveleskie K Tillisch K Kilpatrick L Jarcho J. Sex differences in emotion-related cognitive processes in irritable bowel syndrome and healthy control subjects. Pain. (2013) 154:2088–99. 10.1016/j.pain.2013.06.024 23791896 PMC4049231

[126] Hubbard CS Karpowicz JM Furman AJ da Silva JT Seminowicz DA Traub RJ. Estrogen-dependent visceral hypersensitivity following stress in rats: an fMRI study. Mol Pain. (2016) 12:1744806916654145. 10.1177/1744806916654145 27317579 PMC4956385

[127] Friedrich T Schalla MA Lommel R Goebel-Stengel M Kobelt P Rose M. Restraint stress increases the expression of phoenixin immunoreactivity in rat brain nuclei. Brain Res. (2020) 1743:146904. 10.1016/j.brainres.2020.146904 32474019

[128] Goebel M Stengel A Wang L Taché Y. Restraint stress activates nesfatin-1-immunoreactive brain nuclei in rats. Brain Res. (2009) 1300:114–24. 10.1016/j.brainres.2009.08.082 19733157 PMC2783213

[129] Schalla MA Stengel A. The role of phoenixin in behavior and food intake. Peptides. (2019) 114:38–43. 10.1016/j.peptides.2019.04.002 30953667

[130] Weibert E Hofmann T Stengel A. Role of nesfatin-1 in anxiety, depression and the response to stress. Psychoneuroendocrinology. (2019) 100:58–66. 10.1016/j.psyneuen.2018.09.037 30292960

[131] Foo KS Brismar H Broberger C. Distribution and neuropeptide coexistence of nucleobindin-2 mRNA/nesfatin-like immunoreactivity in the rat CNS. Neuroscience. (2008) 156:563–79. 10.1016/j.neuroscience.2008.07.054 18761059

[132] Yoshida N Maejima Y Sedbazar U Ando A Kurita H Damdindorj B. Stressor-responsive central nesfatin-1 activates corticotropin-releasing hormone, noradrenaline and serotonin neurons and evokes hypothalamic-pituitary-adrenal axis. Aging. (2010) 2:775–84. 10.18632/aging.100207 20966530 PMC3006020

[133] Wang L Gourcerol G Yuan PQ Wu SV Million M Larauche M. Peripheral peptide YY inhibits propulsive colonic motor function through Y2 receptor in conscious mice. Am J Physiol Gastrointest Liver Physiol. (2010) 298:G45–56. 10.1152/ajpgi.00349.2009 19892938 PMC2806102

[134] Ait-Belgnaoui A Han W Lamine F Eutamene H Fioramonti J Bueno L. Lactobacillus farciminis treatment suppresses stress induced visceral hypersensitivity: a possible action through interaction with epithelial cell cytoskeleton contraction. Gut. (2006) 55:1090–4. 10.1136/gut.2005.084194 16507583 PMC1856261

[135] Traini C Evangelista S Girod V Faussone-Pellegrini MS Vannucchi MG. Changes of excitatory and inhibitory neurotransmitters in the colon of rats underwent to the wrap partial restraint stress. Neurogastroenterol Motil. (2016) 28:1172–85. 10.1111/nmo.12816 26972279

[136] Boulete IM Thadi A Beaufrand C Patwa V Joshi A Foss JA. Oral treatment with plecanatide or dolcanatide attenuates visceral hypersensitivity via activation of guanylate cyclase-C in rat models. World J Gastroenterol. (2018) 24:1888–900. 10.3748/wjg.v24.i17.1888 29740204 PMC5937206

[137] Nakade Y Tsuchida D Fukuda H Iwa M Pappas TN Takahashi T. Restraint stress delays solid gastric emptying via a central CRF and peripheral sympathetic neuron in rats. Am J Physiol Regul Integr Comp Physiol. (2005) 288:R427–32. 10.1152/ajpregu.00499.2004 15458973

[138] Million M Maillot C Saunders P Rivier J Vale W Tache Y. Human urocortin II, a new CRF-related peptide, displays selective CRF(2)-mediated action on gastric transit in rats. Am J Physiol Gastrointest Liver Physiol. (2002) 282:G34–40. 10.1152/ajpgi.00283.2001 11751155

[139] Seto K Sasaki T Katsunuma K Kobayashi N Tanaka K Tack J. Acotiamide hydrochloride (Z-338), a novel prokinetic agent, restores delayed gastric emptying and feeding inhibition induced by restraint stress in rats. Neurogastroenterol Motil. (2008) 20:1051–9. 10.1111/j.1365-2982.2008.01135.x 18482254

[140] Ye Y Wang XR Zheng Y Yang JW Yang NN Shi GX. Choosing an animal model for the study of functional dyspepsia. Can J Gastroenterol Hepatol. (2018) 2018:1531958. 10.1155/2018/1531958 29623262 PMC5830275

[141] Bliss TV Collingridge GL Kaang BK Zhuo M. Synaptic plasticity in the anterior cingulate cortex in acute and chronic pain. Nat Rev Neurosci. (2016) 17:485–96. 10.1038/nrn.2016.68 27307118

[142] Hostinar CE Sullivan RM Gunnar MR. Psychobiological mechanisms underlying the social buffering of the hypothalamic-pituitary-adrenocortical axis: a review of animal models and human studies across development. Psychol Bull. (2014) 140:256–82. 10.1037/a0032671 23607429 PMC3844011

[143] Levine S Johnson DF Gonzalez CA. Behavioral and hormonal responses to separation in infant rhesus monkeys and mothers. Behav Neurosci. (1985) 99:399–410. 10.1037/0735-7044.99.3.399 3843717

[144] Hennessy MB Kaiser S Sachser N. Social buffering of the stress response: diversity, mechanisms, and functions. Front Neuroendocrinol. (2009) 30:470–82. 10.1016/j.yfrne.2009.06.001 19545584

[145] Lauffer A Vanuytsel T Vanormelingen C Vanheel H Salim Rasoel S Toth J. Subacute stress and chronic stress interact to decrease intestinal barrier function in rats. Stress. (2016) 19:225–34. 10.3109/10253890.2016.1154527 26947111

[146] Vicario M Guilarte M Alonso C Yang P Martinez C Ramos L. Chronological assessment of mast cell-mediated gut dysfunction and mucosal inflammation in a rat model of chronic psychosocial stress. Brain Behav Immun. (2010) 24:1166–75. 10.1016/j.bbi.2010.06.002 20600818

[147] Gadek-Michalska A Tadeusz J Bugajski A Bugajski J. Chronic isolation stress affects subsequent crowding stress-induced brain nitric oxide synthase (NOS) Isoforms and hypothalamic-pituitary-adrenal (HPA) axis responses. Neurotox Res. (2019) 36:523–39. 10.1007/s12640-019-00067-1 31209786 PMC6745034

[148] Berry A Bellisario V Capoccia S Tirassa P Calza A Alleva E. Social deprivation stress is a triggering factor for the emergence of anxiety- and depression-like behaviours and leads to reduced brain BDNF levels in C57BL/6J mice. Psychoneuroendocrinology. (2012) 37:762–72. 10.1016/j.psyneuen.2011.09.007 21974975

[149] Nishida K Kamizato M Kawai T Masuda K Takeo K Teshima-Kondo S. Interleukin-18 is a crucial determinant of vulnerability of the mouse rectum to psychosocial stress. FASEB J. (2009) 23:1797–805. 10.1096/fj.08-125005 19141531

[150] Stanisor OI van Diest SA Yu Z Welting O Bekkali N Shi J. Stress-induced visceral hypersensitivity in maternally separated rats can be reversed by peripherally restricted histamine-1-receptor antagonists. PLoS ONE. (2013) 8:e66884. 10.1371/journal.pone.0066884 23776699 PMC3680390

[151] Prusator DK Greenwood-van Meerveld B. Sex differences in stress-induced visceral hypersensitivity following early life adversity: a two hit model. Neurogastroenterol Motil. (2016) 28:1876–89. 10.1111/nmo.12891 27385091

[152] Jianguo L Xueyang J Cui W Changxin W Xuemei Q Altered gut metabolome contributes to depression-like behaviors in rats exposed to chronic unpredictable mild stress. Transl Psychiatry. (2019) 9:40 10.1038/s41398-019-0645-9 30696813 PMC6351597

[153] Li H Wang P Huang L Li P Zhang D. Effects of regulating gut microbiota on the serotonin metabolism in the chronic unpredictable mild stress rat model. Neurogastroenterol Motil. (2019) 31:e13677. 10.1111/nmo.13677 31323174 PMC6852474

[154] Lee YY Annamalai C Rao SSC Post-infectious irritable bowel syndrome. Curr Gastroenterol Rep. (2017) 19:56 10.1007/s11894-017-0595-4 28948467

[155] Teruel C Garrido E Mesonero F. Diagnosis and management of functional symptoms in inflammatory bowel disease in remission. World J Gastrointest Pharmacol Ther. (2016) 7:78–90. 10.4292/wjgpt.v7.i1.78 26855814 PMC4734957

[156] Klem F Wadhwa A Prokop LJ Sundt WJ Farrugia G Camilleri M. Prevalence, risk factors, and outcomes of irritable bowel syndrome after infectious enteritis: a systematic review and meta-analysis. Gastroenterology. (2017) 152:1042–54.e1. 10.1053/j.gastro.2016.12.039 28069350 PMC5367939

[157] Nozu T Miyagishi S Nozu R Takakusaki K Okumura T. Lipopolysaccharide induces visceral hypersensitivity: role of interleukin-1, interleukin-6, and peripheral corticotropin-releasing factor in rats. J Gastroenterol. (2017) 52:72–80. 10.1007/s00535-016-1208-y 27075754

[158] Coelho AM Fioramonti J Buéno L. Systemic lipopolysaccharide influences rectal sensitivity in rats: role of mast cells, cytokines, and vagus nerve. Am J Physiol Gastrointest Liver Physiol. (2000) 279:G781–90. 10.1152/ajpgi.2000.279.4.G781 11005766

[159] Nozu T Miyagishi S Nozu R Takakusaki K Okumura T. Altered colonic sensory and barrier functions by CRF: roles of TLR4 and IL-1. J Endocrinol. (2018) 239:241–52. 10.1530/JOE-18-0441 30139928

[160] Nozu T Miyagishi S Nozu R Takakusaki K Okumura T Losartan improves visceral sensation and gut barrier in a rat model of irritable bowel syndrome. Neurogastroenterol Motil. (2020) 32:e13819 10.1111/nmo.13819 32056324

[161] Nozu T Miyagishi S Kumei S Nozu R Takakusaki K Okumura T Lovastatin inhibits visceral allodynia and increased colonic permeability induced by lipopolysaccharide or repeated water avoidance stress in rats. Eur J Pharmacol. (2018) 818:228–34. 10.1016/j.ejphar.2017.10.056 29107672

[162] Nozu T Miyagishi S Ishioh M Takakusaki K Okumura T. Imipramine improves visceral sensation and gut barrier in rat models of irritable bowel syndrome. Eur J Pharmacol. (2020) 887:173565. 10.1016/j.ejphar.2020.173565 32946869

[163] Scanzi J Accarie A Muller E Pereira B Aissouni Y Goutte M. Colonic overexpression of the T-type calcium channel Cav 3.2 in a mouse model of visceral hypersensitivity and in irritable bowel syndrome patients. Neurogastroenterol Motil. (2016) 28:1632–40. 10.1111/nmo.12860 27196538

[164] Picard E Carvalho FA Agosti F Bourinet E Ardid D Eschalier A Inhibition of Cav3.2 calcium channels: a new target for colonic hypersensitivity associated with low-grade inflammation. Br J Pharmacol. (2019) 176:950–63. 10.1111/bph.14608 30714145 PMC6433640

[165] Adam B Liebregts T Gschossmann JM Krippner C Scholl F Ruwe M. Severity of mucosal inflammation as a predictor for alterations of visceral sensory function in a rat model. Pain. (2006) 123:179–86. 10.1016/j.pain.2006.02.029 16630696

[166] Tada Y Ishihara S Kawashima K Fukuba N Sonoyama H Kusunoki R. Downregulation of serotonin reuptake transporter gene expression in healing colonic mucosa in presence of remaining low-grade inflammation in ulcerative colitis. J Gastroenterol Hepatol. (2016) 31:1443–52. 10.1111/jgh.13268 26676714

[167] Lapointe TK Basso L Iftinca MC Flynn R Chapman K Dietrich G. TRPV1 sensitization mediates postinflammatory visceral pain following acute colitis. Am J Physiol Gastrointest Liver Physiol. (2015) 309:G87–99. 10.1152/ajpgi.00421.2014 26021808

[168] Qin HY Wu JC Tong XD Sung JJ Xu HX Bian ZX. Systematic review of animal models of post-infectious/post-inflammatory irritable bowel syndrome. J Gastroenterol. (2011) 46:164–74. 10.1007/s00535-010-0321-6 20848144

[169] Johnson AC Greenwood-van Meerveld B. Critical evaluation of animal models of gastrointestinal disorders. Handb Exp Pharmacol. (2017) 239:289–317. 10.1007/164_2016_120 28176046

[170] Fabisiak A Włodarczyk J Fabisiak N Storr M Fichna J. Targeting histamine receptors in irritable bowel syndrome: a critical appraisal. J Neurogastroenterol Motil. (2017) 23:341–8. 10.5056/jnm16203 28551943 PMC5503283

[171] Winston JH Sarna SK. Developmental origins of functional dyspepsia-like gastric hypersensitivity in rats. Gastroenterology. (2013) 144:570–9.e3. 10.1053/j.gastro.2012.11.001 23142231 PMC3578170

[172] Stanghellini V. Functional dyspepsia and irritable bowel syndrome: beyond Rome IV. Dig Dis. (2017) 35(Suppl. 1):14–7. 10.1159/000485408 29421792

[173] Winston J Shenoy M Medley D Naniwadekar A Pasricha PJ. The vanilloid receptor initiates and maintains colonic hypersensitivity induced by neonatal colon irritation in rats. Gastroenterology. (2007) 132:615–27. 10.1053/j.gastro.2006.11.014 17258716

[174] Hou Q Huang Y Zhu S Li P Chen X Hou Z. MiR-144 increases intestinal permeability in IBS-D rats by targeting OCLN and ZO1. Cell Physiol Biochem. (2017) 44:2256–68. 10.1159/000486059 29258088

[175] Dai F Lei Y Li S Song G Chen JD. Desvenlafaxine succinate ameliorates visceral hypersensitivity but delays solid gastric emptying in rats. Am J Physiol Gastrointest Liver Physiol. (2013) 305:G333–9. 10.1152/ajpgi.00224.2012 23764892

[176] Jones RC 3rd Otsuka E Wagstrom E Jensen CS Price MP Gebhart GF. Short-term sensitization of colon mechanoreceptors is associated with long-term hypersensitivity to colon distention in the mouse. Gastroenterology. (2007) 133:184–94. 10.1053/j.gastro.2007.04.042 17553498

[177] Banvolgyi A Pozsgai G Brain SD Helyes ZS Szolcsanyi J Ghosh M. Mustard oil induces a transient receptor potential vanilloid 1 receptor-independent neurogenic inflammation and a non-neurogenic cellular inflammatory component in mice. Neuroscience. (2004) 125:449–59. 10.1016/j.neuroscience.2004.01.009 15062987

[178] Chun E Yoon S Parveen A Jin M. Alleviation of irritable bowel syndrome-like symptoms and control of gut and brain responses with oral administration of Dolichos lablab L. in a mouse model. Nutrients. (2018) 10:1475. 10.3390/nu10101475 30309025 PMC6213091

[179] Ji Y Tang B Cao DY Wang G Traub RJ. Sex differences in spinal processing of transient and inflammatory colorectal stimuli in the rat. Pain. (2012) 153:1965–73. 10.1016/j.pain.2012.06.019 22819535 PMC3413769

[180] Kimball ES Palmer JM D'Andrea MR Hornby PJ Wade PR. Acute colitis induction by oil of mustard results in later development of an IBS-like accelerated upper GI transit in mice. Am J Physiol Gastrointest Liver Physiol. (2005) 288:G1266–73. 10.1152/ajpgi.00444.2004 15691868

[181] Zhang MM Liu SB Chen T Koga K Zhang T Li YQ. Effects of NB001 and gabapentin on irritable bowel syndrome-induced behavioral anxiety and spontaneous pain. Mol Brain. (2014) 7:47. 10.1186/1756-6606-7-47 24935250 PMC4071154

[182] Liu LS Winston JH Shenoy MM Song GQ Chen JD Pasricha PJ. A rat model of chronic gastric sensorimotor dysfunction resulting from transient neonatal gastric irritation. Gastroenterology. (2008) 134:2070–9. 10.1053/j.gastro.2008.02.093 18448102

[183] Heiss CN Olofsson LE. The role of the gut microbiota in development, function and disorders of the central nervous system and the enteric nervous system. J Neuroendocrinol. (2019) 31:e12684. 10.1111/jne.12684 30614568

[184] Spiller R Garsed K. Postinfectious irritable bowel syndrome. Gastroenterology. (2009) 136:1979–88. 10.1053/j.gastro.2009.02.074 19457422

[185] Schwille-Kiuntke J Mazurak N Enck P. Systematic review with meta-analysis: post-infectious irritable bowel syndrome after travellers' diarrhoea. Aliment Pharmacol Ther. (2015) 41:1029–37. 10.1111/apt.13199 25871571

[186] Halliez MC Buret AG. Gastrointestinal parasites and the neural control of gut functions. Front Cell Neurosci. (2015) 9:452. 10.3389/fncel.2015.00452 26635531 PMC4658430

[187] Akiho H Deng Y Blennerhassett P Kanbayashi H Collins SM. Mechanisms underlying the maintenance of muscle hypercontractility in a model of postinfective gut dysfunction. Gastroenterology. (2005) 129:131–41. 10.1053/j.gastro.2005.03.049 16012943

[188] Venkova K Greenwood-van Meerveld B. Long-lasting changes in small intestinal transport following the recovery from *Trichinella spiralis* infection. Neurogastroenterol Motil. (2006) 18:234–42. 10.1111/j.1365-2982.2005.00753.x 16487415

[189] Bercik P Wang L Verdu EF Mao YK Blennerhassett P Khan WI. Visceral hyperalgesia and intestinal dysmotility in a mouse model of postinfective gut dysfunction. Gastroenterology. (2004) 127:179–87. 10.1053/j.gastro.2004.04.006 15236184

[190] Barnich N Carvalho FA Glasser AL Darcha C Jantscheff P Allez M. CEACAM6 acts as a receptor for adherent-invasive *E. coli*, supporting ileal mucosa colonization in Crohn disease. J Clin Invest. (2007) 117:1566–74. 10.1172/JCI30504 17525800 PMC1868786

[191] Lashermes A Boudieu L Barbier J Sion B Gelot A Barnich N. Adherent-Invasive *E. coli* enhances colonic hypersensitivity and P2X receptors expression during post-infectious period. Gut Microbes. (2018) 9:26–37. 10.1080/19490976.2017.1361091 28806140 PMC5914911

[192] Bohn L Storsrud S Tornblom H Bengtsson U Simren M. Self-reported food-related gastrointestinal symptoms in IBS are common and associated with more severe symptoms and reduced quality of life. Am J Gastroenterol. (2013) 108:634–41. 10.1038/ajg.2013.105 23644955

[193] Fritscher-Ravens A Schuppan D Ellrichmann M Schoch S Rocken C Brasch J. Confocal endomicroscopy shows food-associated changes in the intestinal mucosa of patients with irritable bowel syndrome. Gastroenterology. (2014) 147:1012–20.e4. 10.1053/j.gastro.2014.07.046 25083606

[194] Smit JJ Noti M O'Mahony L The use of animal models to discover immunological mechanisms underpinning sensitization to food allergens. Drug Discov Today. (2015) 17–18:63–9. 10.1016/j.ddmod.2016.09.001

[195] Liu T Navarro S Lopata AL. Current advances of murine models for food allergy. Mol Immunol. (2016) 70:104–17. 10.1016/j.molimm.2015.11.011 26759987

[196] Florens M Aguilera-Lizarraga J Theofanous S Bosmans G Balemans D Perna E Food antigen-specific antibodies and mast cell activation in post-infectious visceral hypersensitivity. Gastroenterology. (2017) 152:S721 10.1016/S0016-5085(17)32508-8

[197] Aguilera-Lizarraga J Balemans D Lopez CDL Polanco JOJ Florens M Perna E 1092 - Food antigen-specific sensitization of nociceptive nerves as an underlying mechanism of visceral pain in Ibs. Gastroenterology. (2018) 154:S-214-S-5 10.1016/S0016-5085(18)31107-7

[198] Bouchoucha M Fysekidis M Julia C Airinei G Catheline J-M Reach G. Functional gastrointestinal disorders in obese patients. The importance of the enrollment source. Obes Surg. (2015) 25:2143–52. 10.1007/s11695-015-1679-6 25904236

[199] Aasbrenn M Høgestøl I Eribe I Kristinsson J Lydersen S Mala T. Prevalence and predictors of irritable bowel syndrome in patients with morbid obesity: a cross-sectional study. BMC Obes. (2017) 4:22. 10.1186/s40608-017-0159-z 28680646 PMC5490229

[200] Fysekidis M Bouchoucha M Bihan H Reach G Benamouzig R Catheline JM. Prevalence and co-occurrence of upper and lower functional gastrointestinal symptoms in patients eligible for bariatric surgery. Obes Surg. (2012) 22:403–10. 10.1007/s11695-011-0396-z 21503810

[201] Shau JP Chen PH Chan CF Hsu YC Wu TC James FE. Fast foods–are they a risk factor for functional gastrointestinal disorders? Asia Pac J Clin Nutr. (2016) 25:393–401. 10.6133/apjcn.2016.25.2.28 27222424

[202] Buscail C Sabate JM Bouchoucha M Kesse-Guyot E Hercberg S Benamouzig R. Western dietary pattern is associated with irritable bowel syndrome in the French NutriNet cohort. Nutrients. (2017) 9:986. 10.3390/nu9090986 28880222 PMC5622746

[203] Brun P Castagliuolo I Leo VD Buda A Pinzani M Palù G. Increased intestinal permeability in obese mice: new evidence in the pathogenesis of nonalcoholic steatohepatitis. Am J Physiol Gastrointest Liver Physiol. (2007) 292:G518–25. 10.1152/ajpgi.00024.2006 17023554

[204] Luo Q Cheng D Huang C Li Y Lao C Xia Y. Improvement of colonic immune function with Soy isoflavones in high-fat diet-induced obese rats. Molecules. (2019) 24:1139. 10.3390/molecules24061139 30909396 PMC6470843

[205] Thiennimitr P Yasom S Tunapong W Chunchai T Wanchai K Pongchaidecha A. Lactobacillus paracasei HII01, xylooligosaccharides, and synbiotics reduce gut disturbance in obese rats. Nutrition. (2018) 54:40–7. 10.1016/j.nut.2018.03.005 29705500

[206] Chen M Hou P Zhou M Ren Q Wang X Huang L. Resveratrol attenuates high-fat diet-induced non-alcoholic steatohepatitis by maintaining gut barrier integrity and inhibiting gut inflammation through regulation of the endocannabinoid system. Clin Nutr. (2019) 39:1264–75. 10.1016/j.clnu.2019.05.020 31189495

[207] Ouni M Schurmann A. Epigenetic contribution to obesity. Mamm Genome. (2020) 31:134–45. 10.1007/s00335-020-09835-3 32279091 PMC7368865

[208] Rajilic-Stojanovic M Biagi E Heilig HG Kajander K Kekkonen RA Tims S. Global and deep molecular analysis of microbiota signatures in fecal samples from patients with irritable bowel syndrome. Gastroenterology. (2011) 141:1792–801. 10.1053/j.gastro.2011.07.043 21820992

[209] Bourdu S Dapoigny M Chapuy E Artigue F Vasson MP Dechelotte P. Rectal instillation of butyrate provides a novel clinically relevant model of noninflammatory colonic hypersensitivity in rats. Gastroenterology. (2005) 128:1996–2008. 10.1053/j.gastro.2005.03.082 15940632

[210] Xu D Wu X Grabauskas G Owyang C. Butyrate-induced colonic hypersensitivity is mediated by mitogen-activated protein kinase activation in rat dorsal root ganglia. Gut. (2013) 62:1466–74. 10.1136/gutjnl-2012-302260 22833396 PMC3897301

[211] Long X Li M Li LX Sun YY Zhang WX Zhao DY. Butyrate promotes visceral hypersensitivity in an IBS-like model via enteric glial cell-derived nerve growth factor. Neurogastroenterol Motil. (2018) 30:e13227. 10.1111/nmo.13227 29052293

[212] Nielsen MA Bayati A Mattsson H. Wistar Kyoto rats have impaired gastric accommodation compared to Sprague Dawley rats due to increased gastric vagal cholinergic tone. Scand J Gastroenterol. (2006) 41:773–81. 10.1080/00365520500483215 16785189

[213] Vanuytsel T Vanormelingen C Vanheel H Masaoka T Salim Rasoel S Toth J. From intestinal permeability to dysmotility: the biobreeding rat as a model for functional gastrointestinal disorders. PLoS ONE. (2014) 9:e111132. 10.1371/journal.pone.0111132 25354336 PMC4212994

[214] Mattsson H Arani Z Astin M Bayati A Overstreet DH Lehmann A. Altered neuroendocrine response and gastric dysmotility in the flinders sensitive line rat. Neurogastroenterol Motil. (2005) 17:166–74. 10.1111/j.1365-2982.2005.00665.x 15787937

[215] Like AA Butler L Williams RM Appel MC Weringer EJ Rossini AA Spontaneous autoimmune diabetes mellitus in the BB rat. Diabetes. (1982) 31:7–13. 10.2337/diab.31.1.S7 6761194

[216] Visser JT Lammers K Hoogendijk A Boer MW Brugman S Beijer-Liefers S. Restoration of impaired intestinal barrier function by the hydrolysed casein diet contributes to the prevention of type 1 diabetes in the diabetes-prone BioBreeding rat. Diabetologia. (2010) 53:2621–8. 10.1007/s00125-010-1903-9 20853098 PMC2974912

[217] Neu J Reverte CM Mackey AD Liboni K Tuhacek-Tenace LM Hatch M. Changes in intestinal morphology and permeability in the biobreeding rat before the onset of type 1 diabetes. J Pediatr Gastroenterol Nutr. (2005) 40:589–95. 10.1097/01.MPG.0000159636.19346.C1 15861021

[218] Demedts I Masaoka T Kindt S de Hertogh G Geboes K Farre R. Gastrointestinal motility changes and myenteric plexus alterations in spontaneously diabetic biobreeding rats. J Neurogastroenterol Motil. (2013) 19:161–70. 10.5056/jnm.2013.19.2.161 23667747 PMC3644652

[219] Meleine M Accarie A Wauters L Toth J Gourcerol G Tack J. Colonic hypersensitivity and low-grade inflammation in a spontaneous animal model for functional gastrointestinal disorders. Neurogastroenterol Motil. (2019) 31:e13614. 10.1111/nmo.13614 31069897

[220] Vanormelingen C Vanuytsel T Masaoka T de Hertogh G Vanheel H Vanden Berghe P. The normoglycaemic biobreeding rat: a spontaneous model for impaired gastric accommodation. Gut. (2016) 65:73–81. 10.1136/gutjnl-2014-308154 25410165

[221] Vanuytsel T Di Giovangiulio M Vanormelingen C Vanheel H Rasoel SS Tóth JG Mo2045 the normoglycemic BB-DP rat as a model for functional gastrointestinal disorders: the implication of mast cells and eosinophils. Gastroenterology. (2015) 148:778 10.1016/S0016-5085(15)32654-8

[222] Rittenhouse PA Lopez-Rubalcava C Stanwood GD Lucki I. Amplified behavioral and endocrine responses to forced swim stress in the Wistar-Kyoto rat. Psychoneuroendocrinology. (2002) 27:303–18. 10.1016/S0306-4530(01)00052-X 11818168

[223] Vicario M Alonso C Guilarte M Serra J Martinez C Gonzalez-Castro AM. Chronic psychosocial stress induces reversible mitochondrial damage and corticotropin-releasing factor receptor type-1 upregulation in the rat intestine and IBS-like gut dysfunction. Psychoneuroendocrinology. (2012) 37:65–77. 10.1016/j.psyneuen.2011.05.005 21641728

[224] Hyland NP O'Mahony SM O'Malley D O'Mahony CM Dinan TG Cryan JF. Early-life stress selectively affects gastrointestinal but not behavioral responses in a genetic model of brain-gut axis dysfunction. Neurogastroenterol Motil. (2015) 27:105–13. 10.1111/nmo.12486 25443141

[225] Pare WP Tejani-Butt SM. Effect of stress on the behavior and 5-HT system in Sprague-Dawley and Wistar Kyoto rat strains. Integr Physiol Behav Sci. (1996) 31:112–21. 10.1007/BF02699783 8809595

[226] Scholl JL Renner KJ Forster GL Tejani-Butt S. Central monoamine levels differ between rat strains used in studies of depressive behavior. Brain Res. (2010) 1355:41–51. 10.1016/j.brainres.2010.08.003 20696147 PMC2946061

[227] Gibney SM Gosselin RD Dinan TG Cryan JF. Colorectal distension-induced prefrontal cortex activation in the Wistar-Kyoto rat: implications for irritable bowel syndrome. Neuroscience. (2010) 165:675–83. 10.1016/j.neuroscience.2009.08.076 19765638

[228] Greenwood-Van Meerveld B Johnson AC Cochrane S Schulkin J Myers DA. Corticotropin-releasing factor 1 receptor-mediated mechanisms inhibit colonic hypersensitivity in rats. Neurogastroenterol Motil. (2005) 17:415–22. 10.1111/j.1365-2982.2005.00648.x 15916629

[229] Gunter WD Shepard JD Foreman RD Myers DA Greenwood-van Meerveld B. Evidence for visceral hypersensitivity in high-anxiety rats. Physiol Behav. (2000) 69:379–82. 10.1016/S0031-9384(99)00254-1 10869605

[230] Martinez V Ryttinger M Kjerling M Astin-Nielsen M Characterisation of colonic accommodation in Wistar Kyoto rats with impaired gastric accommodation. Naunyn Schmiedebergs Arch Pharmacol. (2007) 376:205–16. 10.1007/s00210-007-0195-1 17909748

[231] O'Malley D Julio-Pieper M Gibney SM Dinan TG Cryan JF. Distinct alterations in colonic morphology and physiology in two rat models of enhanced stress-induced anxiety and depression-like behaviour. Stress. (2010) 13:114–22. 10.3109/10253890903067418 20214436

[232] Overstreet DH Russell RW. Selective breeding for diisopropyl fluorophosphate-sensitivity: behavioural effects of cholinergic agonists and antagonists. Psychopharmacology. (1982) 78:150–5. 10.1007/BF00432254 6817373

[233] Janowsky DS el-Yousef MK Davis JM Sekerke HJ. A cholinergic-adrenergic hypothesis of mania and depression. Lancet. (1972) 2:632–5. 10.1016/S0140-6736(72)93021-8 4116781

[234] Overstreet DH Wegener G. The flinders sensitive line rat model of depression−25 years and still producing. Pharmacol Rev. (2013) 65:143–55. 10.1124/pr.111.005397 23319547

[235] Tillmann S Abildgaard A Winther G Wegener G. Altered fecal microbiota composition in the flinders sensitive line rat model of depression. Psychopharmacology. (2019) 236:1445–57. 10.1007/s00213-018-5094-2 30470860 PMC6599185

[236] Tillmann S Wegener G. Probiotics reduce risk-taking behavior in the elevated plus maze in the flinders sensitive line rat model of depression. Behav Brain Res. (2019) 359:755–62. 10.1016/j.bbr.2018.08.025 30170032

[237] Wolthuis AM Bislenghi G Fieuws S de Buck van Overstraeten A Boeckxstaens G D'Hoore A. Incidence of prolonged postoperative ileus after colorectal surgery: a systematic review and meta-analysis. Colorectal Dis. (2016) 18:1–9. 10.1111/codi.13210 26558477

[238] Tan S Yu W Lin Z Chen Q Shi J Dong Y. Peritoneal air exposure elicits an intestinal inflammation resulting in postoperative ileus. Mediators Inflamm. (2014) 2014:924296. 10.1155/2014/924296 25140117 PMC4129966

[239] Lyu JH Lee H-T. Effects of dried Citrus unshiu peels on gastrointestinal motility in rodents. Arch Pharm Res. (2013) 36:641–8. 10.1007/s12272-013-0080-z 23463336

[240] Goetz B Benhaqi P Müller MH Kreis ME Kasparek MS. Changes in beta-adrenergic neurotransmission during postoperative ileus in rat circular jejunal muscle. Neurogastroenterol Motil. (2013) 25:154–84. 10.1111/nmo.12020 23009554

[241] Snoek SA Dhawan S van Bree SH Cailotto C van Diest SA Duarte JM Mast cells trigger epithelial barrier dysfunction, bacterial translocation and postoperative ileus in a mouse model. Neurogastroenterol Motil. (2012) 24:172–91. 10.1111/j.1365-2982.2011.01820.x 22122661

[242] Gomez-Pinilla PJ Farro G Di Giovangiulio M Stakenborg N Nemethova A de Vries A. Mast cells play no role in the pathogenesis of postoperative ileus induced by intestinal manipulation. PLoS ONE. (2014) 9:e85304. 10.1371/journal.pone.0085304 24416383 PMC3887017

[243] Stoffels B Hupa KJ Snoek SA van Bree S Stein K Schwandt T. Postoperative ileus involves interleukin-1 receptor signaling in enteric glia. Gastroenterology. (2014) 146:176–87.e1. 10.1053/j.gastro.2013.09.030 24067878

[244] Matteoli G Gomez-Pinilla PJ Nemethova A Di Giovangiulio M Cailotto C van Bree SH. A distinct vagal anti-inflammatory pathway modulates intestinal muscularis resident macrophages independent of the spleen. Gut. (2014) 63:938–48. 10.1136/gutjnl-2013-304676 23929694

[245] The FO Boeckxstaens GE Snoek SA Cash JL Bennink R LaRosa GJ. Activation of the cholinergic anti-inflammatory pathway ameliorates postoperative ileus in mice. Gastroenterology. (2007) 133:1219–28. 10.1053/j.gastro.2007.07.022 17919496

[246] Stakenborg N Wolthuis AM Gomez-Pinilla PJ Farro G Di Giovangiulio M Bosmans G. Abdominal vagus nerve stimulation as a new therapeutic approach to prevent postoperative ileus. Neurogastroenterol Motil. (2017) 29:e13075. 10.1111/nmo.13075 28429863

[247] Taché Y Yang H Miampamba M Martinez V Yuan PQ. Role of brainstem TRH/TRH-R1 receptors in the vagal gastric cholinergic response to various stimuli including sham-feeding. Auton Neurosci. (2006) 125:42–52. 10.1016/j.autneu.2006.01.014 16520096 PMC8086327

[248] Falkén Y Webb DL Abraham-Nordling M Kressner U Hellström PM Näslund E. Intravenous ghrelin accelerates postoperative gastric emptying and time to first bowel movement in humans. Neurogastroenterol Motil. (2013) 25:474–80. 10.1111/nmo.12098 23527561

[249] Luckey A Wang L Jamieson PM Basa NR Million M Czimmer J. Corticotropin-releasing factor receptor 1-deficient mice do not develop postoperative gastric ileus. Gastroenterology. (2003) 125:654–9. 10.1016/S0016-5085(03)01069-2 12949710

[250] Zittel TT Lloyd KC Rothenhöfer I Wong H Walsh JH Raybould HE. Calcitonin gene-related peptide and spinal afferents partly mediate postoperative colonic ileus in the rat. Surgery. (1998) 123:518–27. 10.1067/msy.1998.88090 9591004

[251] Stengel A Goebel-Stengel M Wang L Shaikh A Lambrecht NW Rivier J. Abdominal surgery inhibits circulating acyl ghrelin and ghrelin-O-acyltransferase levels in rats: role of the somatostatin receptor subtype 2. Am J Physiol Gastrointest Liver Physiol. (2011) 301:G239–48. 10.1152/ajpgi.00018.2011 21636529 PMC3154605

[252] Stengel A Goebel M Wang L Taché Y. Abdominal surgery activates nesfatin-1 immunoreactive brain nuclei in rats. Peptides. (2010) 31:263–70. 10.1016/j.peptides.2009.11.015 19944727 PMC3166548

[253] Greenwood-Van Meerveld B Gibson M Gunter W Shepard J Foreman R Myers D. Stereotaxic delivery of corticosterone to the amygdala modulates colonic sensitivity in rats. Brain Res. (2001) 893:135–42. 10.1016/S0006-8993(00)03305-9 11223001

[254] Johnson AC Greenwood-van Meerveld B. Knockdown of steroid receptors in the central nucleus of the amygdala induces heightened pain behaviors in the rat. Neuropharmacology. (2015) 93:116–23. 10.1016/j.neuropharm.2015.01.018 25656477 PMC4929985

[255] Johnson AC Tran L Greenwood-Van Meerveld B. Knockdown of corticotropin-releasing factor in the central amygdala reverses persistent viscerosomatic hyperalgesia. Transl Psychiatry. (2015) 5:e517. 10.1038/tp.2015.16 25734510 PMC4354346

[256] Tran L Schulkin J Ligon CO Greenwood-van Meerveld B. Epigenetic modulation of chronic anxiety and pain by histone deacetylation. Mol Psychiatry. (2014) 20:1219–31. 10.1038/mp.2014.122 25288139

[257] Sekiguchi F Tsubota M Kawabata A. Involvement of voltage-gated calcium channels in inflammation and inflammatory pain. Biol Pharm Bull. (2018) 41:1127–34. 10.1248/bpb.b18-00054 30068860

[258] Holzer P. Acid-sensing ion channels in gastrointestinal function. Neuropharmacology. (2015) 94:72–9. 10.1016/j.neuropharm.2014.12.009 25582294 PMC4458375

[259] Kawabata A Kawao N Kitano T Matsunami M Satoh R Ishiki T. Colonic hyperalgesia triggered by proteinase-activated receptor-2 in mice: involvement of endogenous bradykinin. Neurosci Lett. (2006) 402:167–72. 10.1016/j.neulet.2006.03.074 16644120

[260] Park MK Cho MK Kang SA Park H-K Kim YS Kim KU. Protease-activated receptor 2 is involved in Th2 responses against *Trichinella spiralis* infection. Korean J Parasitol. (2011) 49:235–43. 10.3347/kjp.2011.49.3.235 22072823 PMC3210840

[261] Yuan B Tang W-H Lu L-J Zhou Y Zhu H-Y Zhou Y-L. TLR4 upregulates CBS expression through NF-κB activation in a rat model of irritable bowel syndrome with chronic visceral hypersensitivity. World J Gastroenterol. (2015) 21:8615–28. 10.3748/wjg.v21.i28.8615 26229403 PMC4515842

[262] McKernan DP Gaszner G Quigley EM Cryan JF Dinan TG. Altered peripheral toll-like receptor responses in the irritable bowel syndrome. Aliment Pharmacol Ther. (2011) 33:1045–52. 10.1111/j.1365-2036.2011.04624.x 21453321

[263] Brint EK MacSharry J Fanning A Shanahan F Quigley EMM. Differential expression of Toll-like receptors in patients with irritable bowel syndrome. American J Gastroenterol. (2011) 106:329–36. 10.1038/ajg.2010.438 21102570

[264] Tang H-L Zhang G Ji N-N Du L Chen B-B Hua R. Toll-like receptor 4 in paraventricular nucleus mediates visceral hypersensitivity induced by maternal separation. Front Pharmacol. (2017) 8:309. 10.3389/fphar.2017.00309 28611665 PMC5447361

[265] Bielefeldt K Lamb K Gebhart GF. Convergence of sensory pathways in the development of somatic and visceral hypersensitivity. Am J Physiol Gastrointest Liver Physiol. (2006) 291:G658–65. 10.1152/ajpgi.00585.2005 16500917

[266] Ustinova EE Fraser MO Pezzone MA. Colonic irritation in the rat sensitizes urinary bladder afferents to mechanical and chemical stimuli: an afferent origin of pelvic organ cross-sensitization. Am J Physiol Renal Physiol. (2006) 290:F1478–87. 10.1152/ajprenal.00395.2005 16403832

[267] Greenwood-van Meerveld B Mohammadi E Tyler K van Gordon S Parker A Towner R. Mechanisms of visceral organ crosstalk: importance of alterations in permeability in rodent models. J Urol. (2015) 194:804–11. 10.1016/j.juro.2015.02.2944 25776913 PMC4699674

[268] Winnard KP Dmitrieva N Berkley KJ. Cross-organ interactions between reproductive, gastrointestinal, and urinary tracts: modulation by estrous stage and involvement of the hypogastric nerve. Am J Physiol Regul Integr Comp Physiol. (2006) 291:R1592–601. 10.1152/ajpregu.00455.2006 16946082

[269] Hussain Z Jung DH Lee YJ Park H. The effect of Trimebutine on the overlap syndrome model of guinea pigs. J Neurogastroenterol Motil. (2018) 24:669–75. 10.5056/jnm18049 30114898 PMC6175557

[270] Hussain Z Kim HW Huh CW Lee YJ Park H. The effect of peripheral CRF peptide and water avoidance stress on colonic and gastric transit in guinea pigs. Yonsei Med J. (2017) 58:872–7. 10.3349/ymj.2017.58.4.872 28541004 PMC5447122

[271] Park JJ Chon NR Lee YJ Park H. The effects of an extract of atractylodes Japonica Rhizome, SKI3246 on gastrointestinal motility in guinea pigs. J Neurogastroenterol Motil. (2015) 21:352–60. 10.5056/jnm14112 26130631 PMC4496911

[272] Tanaka T Tanaka A Nakamura A Matsushita K Imanishi A Matsumoto-Okano S. Effects of TAK-480, a novel tachykinin NK(2)-receptor antagonist, on visceral hypersensitivity in rabbits and ricinoleic acid-induced defecation in guinea pigs. J Pharmacol Sci. (2012) 120:15–25. 10.1254/jphs.12085FP 22893394

[273] Brown DR Timmermans JP. Lessons from the porcine enteric nervous system. Neurogastroenterol Motil. (2004) 16:50–4. 10.1111/j.1743-3150.2004.00475.x 15066005

[274] Timmermans J-P Hens J Adriaensen D. Outer submucous plexus: an intrinsic nerve network involved in both secretory and motility processes in the intestine of large mammals and humans. Anat Rec. (2001) 262:71–8. 10.1002/1097-0185(20010101)262:1<;71::AID-AR1012>;3.0.CO;2-A 11146430

[275] Gieling ET Schuurman T Nordquist RE van der Staay FJ. The pig as a model animal for studying cognition and neurobehavioral disorders. In: Hagan JJ, editor. Molecular and Functional Models in Neuropsychiatry. Berlin, Heidelberg: Springer Berlin Heidelberg (2011). p. 359–83. 10.1007/7854_2010_112 21287323

[276] Medland JE Pohl CS Edwards LL Frandsen S Bagley K Li Y. Early life adversity in piglets induces long-term upregulation of the enteric cholinergic nervous system and heightened, sex-specific secretomotor neuron responses. Neurogastroenterol Motil. (2016) 28:1317–29. 10.1111/nmo.12828 27134125 PMC5002263

[277] Moeser AJ Ryan KA Nighot PK Blikslager AT. Gastrointestinal dysfunction induced by early weaning is attenuated by delayed weaning and mast cell blockade in pigs. Am J Physiol Gastrointest Liver Physiol. (2007) 293:G413–21. 10.1152/ajpgi.00304.2006 17525151

[278] Smith F Clark JE Overman BL Tozel CC Huang JH Rivier JE. Early weaning stress impairs development of mucosal barrier function in the porcine intestine. Am J Physiol Gastrointest Liver Physiol. (2010) 298:G352–63. 10.1152/ajpgi.00081.2009 19926814 PMC2838512

[279] Moeser AJ Klok CV Ryan KA Wooten JG Little D Cook VL. Stress signaling pathways activated by weaning mediate intestinal dysfunction in the pig. Am J Physiol Gastrointest Liver Physiol. (2007) 292:G173–81. 10.1152/ajpgi.00197.2006 16901995

[280] Overman EL Rivier JE Moeser AJ. CRF induces intestinal epithelial barrier injury via the release of mast cell proteases and TNF-α. PLoS ONE. (2012) 7:e39935. 10.1371/journal.pone.0039935 22768175 PMC3386952

[281] Clayton JA. Studying both sexes: a guiding principle for biomedicine. FASEB J. (2016) 30:519–24. 10.1096/fj.15-279554 26514164 PMC4714546

[282] Miller LR Marks C Becker JB Hurn PD Chen WJ Woodruff T. Considering sex as a biological variable in preclinical research. FASEB J. (2017) 31:29–34. 10.1096/fj.201600781r 27682203 PMC6191005

